# Targeting Mannitol Metabolism as an Alternative Antimicrobial Strategy Based on the Structure-Function Study of Mannitol-1-Phosphate Dehydrogenase in Staphylococcus aureus

**DOI:** 10.1128/mBio.02660-18

**Published:** 2019-07-09

**Authors:** Thanh Nguyen, Truc Kim, Hai Minh Ta, Won Sik Yeo, Jongkeun Choi, Pushpak Mizar, Seung Seo Lee, Taeok Bae, Akhilesh Kumar Chaurasia, Kyeong Kyu Kim

**Affiliations:** a Department of Molecular Cell Biology, Institute for Antimicrobial Research and Therapeutics, Sungkyunkwan University School of Medicine, Suwon, South Korea; b Department of Microbiology and Immunology, Indiana University School of Medicine Northwest, Gary, Indiana, USA; c Department of Chemical Engineering, Chungwoon University, Incheon, South Korea; d School of Chemistry, University of Southampton, Southampton, United Kingdom; e Samsung Biomedical Research Institute, Samsung Advanced Institute for Health Sciences and Technology, Samsung Medical Center, Sungkyunkwan University School of Medicine, Seoul, South Korea; New York University School of Medicine

**Keywords:** mannitol-1-phosphate dehydrogenase, *Staphylococcus aureus*, antibiotic target, antimicrobial resistance, crystal structure, inhibitor, mannitol, virulence

## Abstract

Mannitol-1-phosphate dehydrogenase (M1PDH) is a key enzyme in Staphylococcus aureus mannitol metabolism, but its roles in pathophysiological settings have not been established. We performed comprehensive structure-function analysis of M1PDH from S. aureus USA300, a strain of community-associated methicillin-resistant S. aureus, to evaluate its roles in cell viability and virulence under pathophysiological conditions. On the basis of our results, we propose M1PDH as a potential antibacterial target. *In vitro* cell viability assessment of Δ*mtlD* knockout and complemented strains confirmed that M1PDH is essential to endure pH, high-salt, and oxidative stress and thus that M1PDH is required for preventing osmotic burst by regulating pressure potential imposed by mannitol. The mouse infection model also verified that M1PDH is essential for bacterial survival during infection. To further support the use of M1PDH as an antibacterial target, we identified dihydrocelastrol (DHCL) as a competitive inhibitor of S. aureus M1PDH (*Sa*M1PDH) and confirmed that DHCL effectively reduces bacterial cell viability during host infection. To explain physiological functions of *Sa*M1PDH at the atomic level, the crystal structure of *Sa*M1PDH was determined at 1.7-Å resolution. Structure-based mutation analyses and DHCL molecular docking to the *Sa*M1PDH active site followed by functional assay identified key residues in the active site and provided the action mechanism of DHCL. Collectively, we propose *Sa*M1PDH as a target for antibiotic development based on its physiological roles with the goals of expanding the repertory of antibiotic targets to fight antimicrobial resistance and providing essential knowledge for developing potent inhibitors of *Sa*M1PDH based on structure-function studies.

## Abstract (precis)

Due to the shortage of effective antibiotics against drug-resistant Staphylococcus aureus, new targets are urgently required to develop next-generation antibiotics. We investigated mannitol-1-phosphate dehydrogenase of S. aureus USA300 (*Sa*M1PDH), a key enzyme regulating intracellular mannitol levels, and explored the possibility of using *Sa*M1PDH as a target for developing antibiotic. Since mannitol is necessary for maintaining the cellular redox and osmotic potential, the homeostatic imbalance caused by treatment with a *Sa*M1PDH inhibitor or knockout of the gene encoding *Sa*M1PDH results in bacterial cell death through oxidative and/or mannitol-dependent cytolysis. We elucidated the molecular mechanism of *Sa*M1PDH and the structural basis of substrate and inhibitor recognition by enzymatic and structural analyses of *Sa*M1PDH. Our results strongly support the concept that targeting of *Sa*M1PDH represents an alternative strategy for developing a new class of antibiotics that cause bacterial cell death not by blocking key cellular machinery but by inducing cytolysis and reducing stress tolerance through inhibition of the mannitol pathway.

## INTRODUCTION

Many antibiotics that have been used for the treatment of Staphylococcus aureus infections are becoming ineffective because of the development of antimicrobial resistance (AMR) (1–4). The emergence of AMR in S. aureus strains necessitates identifying novel antibiotic targets and the corresponding interacting drug molecules. In general, a significant number of antibiotics inhibit the key cellular machinery by targeting the components associated with replication, transcription, and protein translation (5). On the other hand, β-lactam antibiotics target bacterial cell wall biosynthesis (6). The cell wall is important for governing bacterial shape, size, and integrity by regulating the pressure potential (Ψ_P_) and mechanochemical properties of cells (7). The solute (organic and inorganic) homeostasis system is also responsible for maintaining cellular integrity by counteracting external osmotic pressure against various forms of pathophysiological stresses (8, 9). Accordingly, switching between the anabolism and catabolism of organic solutes is closely linked to stress adaptation and virulence (9).

**FIG 1:**
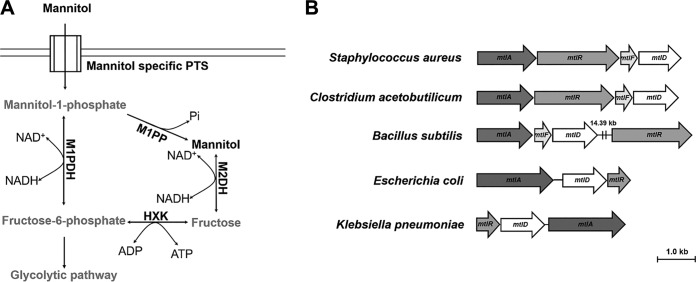
Mannitol metabolism pathway and the mannitol operon in bacteria. (A) A conventional mannitol metabolism pathway. (Adapted from reference 18 with permission.) Extracellular mannitol is taken up by a membrane anchoring the mannitol-specific phosphotransferase system (PTS) as M1P. M1P is then either dephosphorylated by mannitol-1-phosphate phosphatase (M1PP) to generate an intracellular mannitol pool in the bacterial cells or converted to F6P by NAD^+^-dependent oxidase activity of M1PDH. F6P can be either directed to the glycolytic pathway or dephosphorylated by hexokinase (HXK) to generate fructose. Fructose is then converted to mannitol by the NADH-dependent reductase activity of M2DH. In the reverse reaction, F6P is converted to M1P by NADH-dependent reductase activity of M1PDH. M1P is further dephosphorylated by M1PP to generate mannitol stored in the cell to act as a compatible solute. Intracellular mannitol can be converted to fructose by the NAD^+^-dependent oxidase activity of M2DH and further phosphorylated by HXK to generate F6P, which enters the glycolytic pathway. (B) The occurrence of the mannitol operon in a wide range of bacterial genera. Data show the organization of the mannitol operon in Staphylococcus aureus (NC_007793.1), Bacillus subtilis (CP020102.1), Clostridium acetobutylicum (U53868), Escherichia coli (U00096.3), and Klebsiella pneumoniae (FO834906.1).

Intracellular mannitol is the product of glucose metabolism and has been reported to play essential roles as a compatible solute in osmoprotection in bacteria Gluconobacter oxydans, Pseudomonas putida, Acinetobacter baylyi, and Staphylococcus aureus (10–13). Mannitol is one of the most abundant examples of a six-carbon polyol, which controls osmotic pressure and cellular redox (e.g., reactive oxygen species [ROS]) in plants and fungi (14, 15). The intracellular mannitol level is known to influence heat and osmotic stress tolerance, virulence, and pathogenicity in fungal pathogens (16–18). The mannitol pathway is present in plants, fungi, and some bacteria (14, 19, 20). However, studies on such an “intracellular mannitol pool” and its direct correlation with “osmoprotection, ROS tolerance, and virulence” in bacteria are lacking. Recently, the role of mannitol in potentiation of antimicrobial lipid (21) and of gentamicin in effective eradication of methicillin-resistant S. aureus (22) has been reported. From these points of views, the dysregulation of the pressure potential (Ψ_P_) or mechanochemical properties of bacteria through hampering auxiliary metabolic pathways such as the mannitol pathway seems to be an attractive strategy to develop next-generation antibiotics. The mannitol metabolism cycle mediates the interconversion of fructose-6-phosphate (F6P) to mannitol and vice versa in the glycolytic pathway (13). Conventionally, F6P is converted to mannitol-1-phosphate (M1P) by the NADH-dependent mannitol-1-phosphate dehydrogenase (M1PDH), and then M1P is dephosphorylated to mannitol by the M1P phosphatase (M1PP), which is necessary for maintaining osmotic pressure. In the reverse reaction, NAD^+^-dependent mannitol-2-dehydrogenase (M2DH) converts mannitol to fructose, which is then phosphorylated by fructokinase to generate F6P to enter into energy metabolism (15) (Fig. 1A).

In bacteria, the *mtl* operon commonly consists of three or four genes, namely, *mtlA*, *mtlR*, *mtlF*, and *mtlD*, coding for phosphoenolpyruvate-dependent mannitol-specific phosphotransferase system (PTS) enzyme II BC component, a transcriptional activator, an PTS enzyme II A component, and a mannitol dehydrogenase, respectively (Fig. 1B). Thus, *mtlD* is the only gene coding for a single mannitol dehydrogenase enzyme, M1PDH or M2DH, suggesting that the mannitol pathways in bacteria do not follow the conventional mannitol pathway that is present in various plants and fungi (14, 19, 20). M2DHs from many bacteria have been studied for their enzymatic activity, biochemical substrates, and structure (23–25). S. aureus USA300 possesses an M1PDH instead of M2DH. However, the functional role of mannitol metabolism in bacterial pathogenesis and the validity of the mannitol pathway as an antibacterial target have not been established and remain to be explored. Furthermore, due to the absence of M2DH in S. aureus, S. aureus M1PDH (*Sa*M1PDH) appears to be the only enzyme that converts F6P to M1P and vice versa (Fig. 1B); thus, mannitol metabolism in S. aureus is expected to differ significantly from the conventional pathway. From this point of view, we hypothesized that *Sa*M1PDH might have prominent, novel biochemical and physiological roles in S. aureus pathogenicity.

In this study, we established the status of *Sa*M1PDH as a new antibacterial target by verifying that a knockout strain of S. aureus USA300 lacking the functional gene encoding M1PDH (*mtlD*) was cytolyzed by mannitol treatment and showed a reduction in stress tolerance and virulence. We also assessed the mechanistic details of how the intracellular mannitol level is modulated by *Sa*M1PDH in response to extracellular pH, osmotic, and oxidative stresses, which is essential for the pathogen’s defense against stresses imposed by host during infection. Furthermore, we identified the small-molecule inhibitor of *Sa*M1PDH that effectively reduced the survival of S. aureus USA300 in macrophages. The crystal structure of *Sa*M1PDH, solved at 1.7-Å resolution, revealed critical residues for substrate recognition and established structural models for substrate and inhibitor binding. Taking the results together, this report not only provides a description of the molecular basis of the structure, reaction mechanism, substrate specificity, and pathophysiological functions of *Sa*M1PDH but also identifies a potent inhibitor of M1PDH. These results contribute to a new approach to eradication of MRSA infections.

## RESULTS

### Stress modulation by M1PDH in S. aureus USA300.

**FIG 2:**
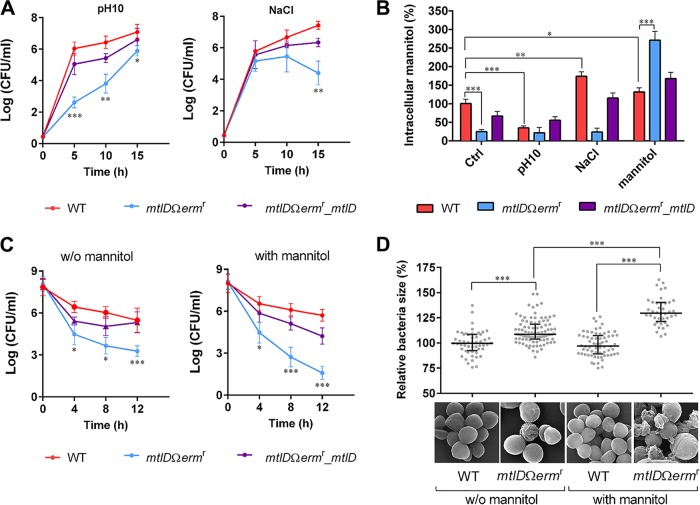
Stress modulation by M1PDH in S. aureus USA300. (A) Effects of abiotic stresses (pH and salt) on the growth of S. aureus USA300 strains. Bacterial growth was assessed by CFU assay at 37°C for 15 h in (left) BHI media adjusted to a pH of 10.0 and (right) BHI media supplemented with 0.2 M NaCl in 96-well plates. (B) Accumulations of intracellular mannitol in S. aureus USA300 strains under various stress conditions. The indicated bacterial strains were cultured in either BHI media (control [Ctrl]) or modified BHI media mimicking stress conditions (pH 10.0, 0.2 M NaCl, and 27.5 mM mannitol). Intracellular mannitol was then extracted from 100 mg of cell pellets and quantified, and levels were calculated as percentages in S. aureus USA300 strains grown under various conditions compared to that of the WT strain grown in control media. (C) Effect of mannitol on Triton X-100-induced autolysis of S. aureus USA300 strains. After cells were cultured in either (left) BHI media or (right) BHI media containing 27.5 mM mannitol, bacterial cell densities of all strains were adjusted to an OD_600_ of 0.9 in PBS containing 0.05% Triton X-100, and survival of each strain was monitored at room temperature for 12 h by the CFU assay. (D) Effect of mannitol on the cell size of S. aureus USA300 examined by scanning electron microscope (SEM). After being cultured at 37°C in either BHI media or BHI media containing 5 mM mannitol for 48 h, WT and *Sa*M1PDH knockout (*mtlD*Ω*erm*^r^) S. aureus USA300 strains were harvested, processed, and visualized under a SEM. From SEM photographs, 40 to 100 cells from each strain were randomly selected for diameter measurements. SEM photographs are shown under a graph of relative bacterial cell diameters versus S. aureus strains with or without mannitol treatment. For the graph in panel D, data are presented as medians (horizontal lines) with interquartile ranges (whiskers), whereas for the other graphs, data are presented as means ± standard deviations of the means. Statistical significance was calculated by Student's *t* test (*, *P < *0.05; **, *P < *0.01; ***, *P < *0.001).

Under pathophysiological conditions, S. aureus experiences various stresses. To understand the functional role of M1PDH in S. aureus (*Sa*M1PDH) under these conditions, we compared the physiologies of wild-type (WT) S. aureus USA300, the corresponding mutant strain (*mtlD*Ω*erm* resistance [*mtlD*Ω*erm*^r^] mutant) lacking the functional gene encoding M1PDH (*mtlD*), and a mutant complemented strain (*mtlD*Ω*erm*^r^_*mtlD*) under normal, high-salt, and alkaline pH stress conditions. Under normal growth conditions in brain heart infusion (BHI) media, these strains showed similar growth profiles (see Fig. S1 in the supplemental material). Intriguingly, the *mtlD*Ω*erm*^r^ strain showed significant growth defects under both pH and salt stress conditions, whereas the corresponding complemented strain overcame such growth defects (Fig. 2A; see also Fig. S1).

10.1128/mBio.02660-18.2 FIG S1 Growth profiles of S. aureus strains under control physiological and various stress conditions. Download FIG S1, PDF file, 0.1 MB. Copyright © 2019 Nguyen et al. 2019 Nguyen et al. This content is distributed under the terms of the Creative Commons Attribution 4.0 International license.

To corroborate the physiological data, we further investigated *mtlD* expression levels under conditions of mannitol, pH, and salt stresses and *mtlD* cotranscription with genes coding for the mannitol*-*specific PTS (*mtlA* and *mtlF*) and verified the phenotypic traits of PTS mutants mimicking the susceptibility of the *mtlDΩerm*^r^ mutant. First, we performed quantitative reverse transcription PCR (qRT-PCR) to assess the levels of expression of individual genes involved in mannitol metabolism (*mtlA*, *mtlR*, *mtlF*, and *mtlD*) under mannitol, salt, and alkaline stress conditions (Fig. S2A). As expected, the supplementation of mannitol induced the expression of *mtlD* by 1.8-fold (Fig. S2A). The abundance of all transcripts from genes *mtlA*, *mtlR*, *mtlF*, and *mtlD* involved in mannitol metabolism under conditions of salt and alkaline stresses was significantly reduced compared to those of the corresponding controls (Fig. S2A). The change in pH is known to decrease PTS transcript levels significantly in S. aureus (26). Second, we compared the expression level of *mtlD* with that of *mtlA*, *mtlR*, and *mtlF* in individual *mtl*-specific *bursa aurealis* transposon mutants of S. aureus USA300 (27) (Fig. S2B). Expression of genes *mtlA*, *mtlR*, and *mtlF* located upstream of *mtlD* was found to be induced in the *mtlDΩerm*^r^ mutant, while *mtlD* expression was repressed in the individual mutants of *mtlAΩerm*^r^, *mtlRΩerm*^r^, and *mtlFΩerm*^r^ (Fig. S2B). These results showing that expression of *mtlD* correlated with expression of *mtlA*, *mtlR*, and *mtlF* under all tested stress conditions (Fig. S2A) and that *mtlD* transcription was downregulated in the individual mutants (*mtlAΩerm*^r^, *mtlRΩerm*^r^, and *mtlFΩerm*^r^) with mutations in the *mtl* operon suggest that *mtlD* is cotranscribed with the genes corresponding to the *mtl*-specific PTS (*mtlA* and *mtlF*) and its regulator (*mtlR*). In addition, the footprinting data determined previously for the *mtlARFD* operon in Bacillus stearothermophilus (28) confirmed the promoter region. Interestingly, the promoter region of B. stearothermophilus showed high sequence similarities with that of B. subtilis and S. carnosus (28) along with the predicted promoter region of the *mtlARFD* operon in S. aureus USA300 (Fig. S2C).

10.1128/mBio.02660-18.3 FIG S2 Transcriptional profiles of *mtlARFD* in wild type S. aureus USA300 and in isogenic mutants of *bursa aurealis* transposon in individual genes of *mtl operon* for phenotypic assessment of mannitol catabolism and alkalinity stress. Download FIG S2, PDF file, 0.4 MB. Copyright © 2019 Nguyen et al. 2019 Nguyen et al. This content is distributed under the terms of the Creative Commons Attribution 4.0 International license.

Third, we carried out the phenotypic assessment of mannitol PTS mutants using the mannitol catabolism assay and of the alkalinity stress response by CFU enumeration. Both the *mtlAΩerm*^r^ PTS mutant and the *mtlFΩerm*^r^ PTS mutant showed an inability to perform mannitol catabolism, similarly to that observed in the *mtlDΩerm*^r^ mutant (Fig. S2D). Furthermore, to assess the essentiality and role of the individual genes, we supplemented the individual mutants (strains *mtlAΩerm*^r^, *mtlRΩerm*^r^, *mtlFΩerm*^r^, and *mtlDΩerm*^r^) with plasmid pRMC2_*mtlD* encoding wild-type *Sa*M1PDH. Interestingly, only the *mtlFΩerm*^r^ strain supplemented with pRMC2_*mtlD* could recover the mannitol catabolism, though it did so at slightly lower efficiency than was seen with the *mtlDΩerm*^r^ mutant complemented with pRMC2_*mtlD* (Fig. S2D). The recovery of mannitol metabolism shown by the *mtlFΩerm*^r^ mutant supplemented with pRMC2_*mtlD* encoding *Sa*M1PDH enzyme suggests that (i) *mtlA* is the major component of the PTS in S. aureus USA300 and that (ii) *mtlF* appears to be a synonymous redundant gene in the mannitol uptake PTS. The analysis of the *mtl* operon showed that the *mtlF* gene is usually absent in various Gram-negative bacterial species, which could further support the idea of the redundancy of *mtlF* in Gram-positive bacteria (Fig. 1B). To further assess and compare the levels of sensitivity to alkalinity stress, we compared the susceptibility of *mtlAΩerm*^r^ and *mtlFΩerm*^r^ PTS mutants with that of the *mtlDΩerm*^r^ mutant under an alkaline condition (Fig. S2E). Consistent with mannitol metabolism (Fig. S2C), both the *mtlAΩerm*^r^ and *mtlFΩerm*^r^ PTS mutants showed susceptibility similar to that shown by the *mtlDΩerm*^r^ mutant (compared to the WT S. aureus strain) under alkaline stress conditions (Fig. S2E). The comparable levels of survival of the *mtlFΩerm*^r^*_mtlD* strain (i.e., the *mtlFΩerm*^r^ mutant supplemented with *pRMC2_mtlD*) and of the *mtlDΩerm*^r^*_mtlD* strain (*mtlDΩerm*^r^ mutant complemented with *pRMC2_mtlD*) and the WT strain (WT or WT with empty vector *pRMC2*) further demonstrated that the PTS enzyme II BC (MtlA) component is the major component of the mannitol-specific PTS (Fig. S2E). Taking these results together, it was evident that the PTS is essential for extracellular mannitol uptake whereas the M1PDH enzyme plays the key role in mannitol metabolism to modulate and alleviate the stress response in S. aureus USA300.

Since mannitol metabolism is directly linked to cellular pH and osmotic pressure, we compared the relative mannitol levels spectrophotometrically (*A*_412_) in the WT and *Sa*M1PDH knockout strains and the corresponding complemented strains under pH and salt stress conditions (Fig. 2B). In this experiment, we saw that the mannitol level in the knockout strain (*mtlD*Ω*erm*^r^) was lower than those in the WT and complemented strains under control, pH, and salt stress conditions (Fig. 2B), suggesting that mannitol was neither consumed nor synthesized in the *Sa*M1PDH-deficient strain. On the other hand, the intracellular mannitol level was much higher in the WT or complemented strains than in the knockout strain under all tested conditions except mannitol supplementation conditions (Fig. 2B). Intriguingly, during growth in mannitol-supplemented media, the level of mannitol was found to be the highest in the *mtlD*-knockout strain, presumably because the continuous import of mannitol and the accumulated mannitol were not catabolized (Fig. 2B). Consistent with the results analyzed by UV-visible light (UV-Vis) spectrometry, the higher mannitol level in the WT strain than in the mutant strain was further confirmed by gas chromatography-mass spectrometry (Fig. S3A). In addition, the S. aureus strain with no *Sa*M1PDH activity (*mtlD*Ω*erm*^r^) exposed to the mannitol-containing medium showed induction of the *mtl* operon at the transcriptional level (Fig. S2A and B). These results indicate that the accumulation of intracellular mannitol in the *mtlD*Ω*erm*^r^ knockout strain is a synergistic effect of increased mannitol uptake resulting from induced expression of the PTS and of no catabolic activity under conditions of accumulated mannitol due to the absence of *Sa*M1PDH in the *mtlD*Ω*erm*^r^ knockout strain. Hence, it could be seen that *Sa*M1PDH is indeed indispensable for the homeostasis of intracellular mannitol under various stress conditions.

10.1128/mBio.02660-18.4 FIG S3 Assessment of accumulations of intracellular mannitol in S. aureus strains and its impact on colony morphology of various S. aureus USA300 strains under control and stress conditions. Download FIG S3, PDF file, 0.3 MB. Copyright © 2019 Nguyen et al. 2019 Nguyen et al. This content is distributed under the terms of the Creative Commons Attribution 4.0 International license.

### Maintenance of cell wall integrity by M1PDH in S. aureus USA300.

Homeostasis of compatible solutes is known to be related to the turgor and osmotic pressures that control cell shape and wall integrity (9). Thus, the issue was whether the presence of *Sa*M1PDH was necessary for the cell wall integrity through controlling the level of mannitol, one of representative compatible solutes. The *mtlD*Ω*erm*^r^ knockout strain cultured for 48 h on the solid media in the presence of mannitol formed bigger and more translucent colonies than the WT strain (Fig. S3B and C), suggesting that the intracellular accumulation of mannitol possibly affected the colony phenotype and arguably altered the shape, size, and wall integrity of the individual cells. Thus, to explore this result in detail, we investigated the contribution of *Sa*M1PDH to the cell wall strength by comparing the levels of Triton X-100-induced cytolysis of the WT, knockout, and complemented strains. The bacteria cultured in the absence and presence of optimized 27.5 mM mannitol for 48 h were subjected to the cytolysis assay (Fig. 2C; see also Fig. S3D). On the basis of the results of this assay, we confirmed that the cytolysis rates of the WT and complemented strains were lower than that of the knockout strain in the absence of mannitol, suggesting that the cell wall of the mutant strains was much weaker than the cell walls of the WT and complemented strains (Fig. 2C, left; see also Fig. S3D, left). This phenomenon was found to have accelerated when cells were cultured in the mannitol-containing medium (Fig. 2C, right; see also Fig. S3D, right). In contrast, the cytolysis rates of the WT and complemented strains were not significantly changed in the mannitol-containing medium (Fig. 2C; see also Fig. S3D).

The swelling and subsequent cytolysis of the *Sa*M1PDH knockout strain in the presence of mannitol were further examined by visualizing the phenotypic alteration of bacteria using scanning electron microscopy (SEM) (Fig. 2D). For SEM analysis, the WT and knockout strains were cultured for 48 h in BHI media with and without mannitol supplementation. The knockout cells grown in the media without mannitol were approximately 11% bigger than the WT cells, suggesting that the mutant cells were slightly swollen even in the absence of mannitol (Fig. 2D). When the knockout cells were grown in mannitol-containing media, they either burst or were significantly enlarged in size by ∼31% compared to the WT cells grown under identical culture conditions, due to mannitol accumulation and consequent inflow of water (Fig. 2D). Furthermore, we compared the levels of membrane permeability of the WT and *mtlD*Ω*erm*^r^ strains and the corresponding complemented strains without or with mannitol by dual staining using wheat germ agglutinin (WGA)-Alexa Fluor 488 conjugate (green) and propidium iodide (PI) (red). WGA stains the cell wall of S. aureus, while PI specifically enters cells with a damaged cell membrane (29). Consistent with the SEM results, negligible numbers of PI-positive S. aureus cells were observed in all strains without mannitol, suggesting that all three strains (i.e., the WT, *mtlD*Ω*erm*^r^, and mutant complemented strains) were healthy under normal growth conditions (Fig. S3E). In contrast, a significantly high number of S. aureus cells in the *mtlD*Ω*erm*^r^ knockout strain showed PI-positive staining whereas most of the WT and complemented S. aureus cells showed PI-negative staining. These results showed that the membrane integrity of the *mtlD*Ω*erm*^r^ knockout strain lacking *Sa*M1PDH activity was indeed compromised under the conditions that included mannitol supplementation, facilitating cytolysis. Taking these results together, we propose that *Sa*M1PDH plays a key role in maintaining the osmotic pressure and cell wall integrity of S. aureus USA300 by controlling the intracellular mannitol level. Therefore, it can be expected that inhibiting *Sa*M1PDH would cause swelling and mannitol-induced cytolysis.

### Antivirulence phenotypes by targeting M1PDH.

**FIG 3:**
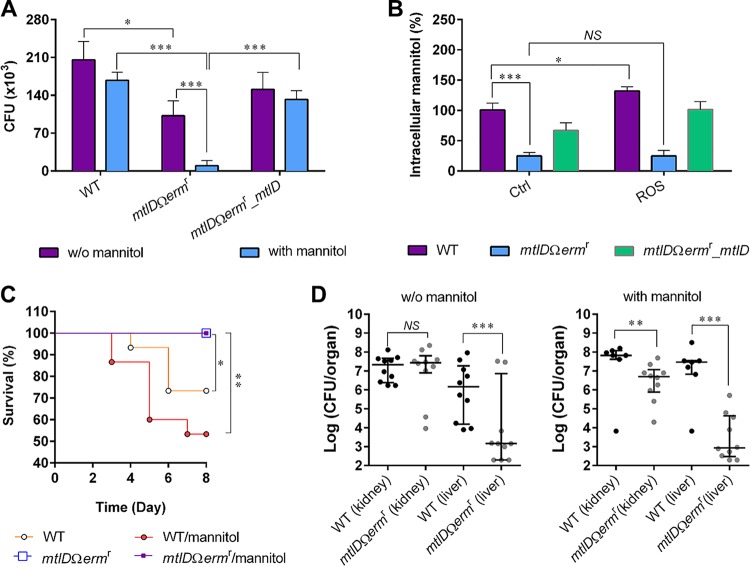
Role of *Sa*M1PDH and effect of mannitol on S. aureus USA300 virulence. (A) Role of *Sa*M1PDH and effect of mannitol on S. aureus USA300 survival rate in macrophages. After infections by WT, *mtlD*Ω*erm*^r^, and *mtlD*Ω*erm*^r^*_mtlD* S. aureus strains followed by removal of extracellular bacteria, infected RAW 264.7 cells were cultured in either control media or media containing 2.75 mM mannitol for 6 h. CFU levels of internalized bacteria were determined by plating infected RAW 264.7 cell lysates onto the BHI agar. (B) Effect of ROS on intracellular mannitol levels in S. aureus USA300 strains. The indicated bacterial strains were cultured in either BHI media (control [Ctrl]) or BHI media containing ROS-generating chemicals (H_2_O_2_, FeSO_4_, and NaI). Intracellular mannitol was then extracted and quantified, and the levels were calculated as percentages of mannitol in S. aureus USA300 strains grown in media containing ROS-generating chemicals compared to that of the WT strain grown in control media. For panels A and B, data were obtained from three independent experiments and are presented as means ± standard deviations of the means. Statistical significance was calculated by Student's *t* test (*, *P < *0.05; ***, *P < *0.001; *NS*, not significant). (C and D) Role of *Sa*M1PDH in and effect of mannitol on pathogenicity of S. aureus USA300 strains in the murine model of systemic infection. Following retro-orbital injection of either WT or knockout (*mtlD*Ω*erm*^r^) S. aureus strains into C57BL/6 mice (*n *=* *15), either PBS or PBS containing mannitol was injected intravenously into these mice at 12-h intervals. (C) A Kaplan Meier plot of mouse survival for each treatment over 8 days was constructed, and statistical significance was calculated by the log rank test (*, *P < *0.05; **, *P < *0.01), Then the surviving mice were subjected to isolation of organs followed by homogenization of the organs in PBS. (D) The colorization of the bacteria in indicated organs was quantified by the CFU assay, and statistical significance was calculated using the *F*-test (*, *P < *0.05; **, *P < *0.01; ***, *P < *0.001).

We demonstrated that M1PDH deficiency in S. aureus USA300 in the presence of mannitol or under salt stress conditions led to bacterial susceptibility and subsequent death (Fig. 2). However, under *in vivo* infection conditions, such a radical change in the mannitol/salt level is unlikely to occur. Nevertheless, even under normal conditions, *Sa*M1PDH deficiency-mediated bacterial susceptibility may help to impede the evasion mechanism of S. aureus USA300. To test this hypothesis, we performed a bacterial infection experiment using the RAW 264.7 mouse macrophage cell line in the absence and presence of mannitol in the culture media (Fig. 3A; see also Fig. S4A and B). RAW 264.7 cells were infected with the WT, knockout, and complementation strains at a multiplicity of infection (MOI) of 100 (30–32). The *in vitro* infection results obtained using modified gentamicin protection assays (GPA) (Fig. 3A) were validated by enzyme protection assays (EPA) (Fig. S4A) wherein the host cell-impermeable lysostaphin enzyme was used as a bacterium-killing agent (33). Enzyme protection assays were applied to avoid any nonspecific killing of intracellular S. aureus due to gentamicin internalization during the GPA (33–35). Compared to that of the WT and complemented strains, the susceptibility of the internalized *mtlD*-knockout strain was found to be significantly increased in both the GPA and EPA (∼50% and ∼34% in the GPA and 56% and ∼46% in the EPA) (Fig. 3A; see also Fig. S4A). Both the lysostaphin-based and gentamicin-based protection assays showed comparative susceptibility results similar to those shown by the respective controls. These results suggested that *Sa*M1PDH plays a significant role in the intracellular survival of S. aureus USA300. This increased susceptibility of the knockout strain was accelerated (∼95% and ∼92% GPA; ∼85% and ∼80% EPA) when cells were treated with 2.75 mM mannitol (Fig. 3A; see also Fig. S4A).

10.1128/mBio.02660-18.5 FIG S4 Role of *Sa*M1PDH with or without mannitol and reactive oxygen species (ROS) in intracellular survival of S. aureus strains assessed by modified enzyme protection assay (EPA); and direct assessment of the differential sensitivities of S. aureus USA300 strains by plate spotting assay. Download FIG S4, PDF file, 0.3 MB. Copyright © 2019 Nguyen et al. 2019 Nguyen et al. This content is distributed under the terms of the Creative Commons Attribution 4.0 International license.

Professional phagocytic cells (macrophages) generate reactive oxygen species (ROS) to kill internalized bacteria by oxidative burst (36). Moreover, mannitol is known to be a powerful scavenger for hydroxyl radicals (37); thus, it plays a vital role for many pathogenic fungi in their defense against host ROS stress (38). In addition, *Sa*M1PDH was reported to help S. aureus overcome hydrogen peroxide (H_2_O_2_) stress (21). We therefore predicted that the increased susceptibility of the internalized knockout strain in RAW 264.7 was the synergistic effect of a weakened ROS defense system and debilitated cell wall. To substantiate this, an infection experiment was performed in the presence of an ROS inhibitor, N-acetyl-l-cysteine (NAC) (Fig. S4B). In this experiment, it was shown that the addition of NAC enhanced intracellular survival of all three S. aureus strains. The survival rates of the WT and complemented strains were increased about 3× whereas the survival of the knockout strain was increased 5×, indicating that the knockout strain was more sensitive to ROS and thus that inhibition by NAC was more pronounced. This suggests that *Sa*M1PDH highly likely contributes to ROS resistance of S. aureus during infection (Fig. S4B). In addition, the survival rates of WT and knockout strains were compared by the dilution spotting assay using BHI agar plates containing a hydroxyl radical generating system (100 μM H_2_O_2_ plus 10 μM FeSO_4_ plus 10 μM NaI) (39) to assess the ROS susceptibility resulting from *Sa*M1PDH deficiency (Fig. S4C). The *Sa*M1PDH knockout strain (*mtlDΩerm*^r^ mutant) was 100-fold more susceptible to ROS stress than the WT S. aureus strain (Fig. S4C). Consistent with our results, susceptibility of the *mtlDΩerm*^r^ mutant under conditions of hydrogen peroxide treatment has previously been reported by Kenny et al. (21). Interestingly, the hydroxyl radical exposure resulted in slight (∼33%) accumulations of mannitol in the WT and complemented strains (Fig. 3B) but not in the knockout strain, which plausibly indicates that ROS treatment enhanced the intracellular mannitol level. These results suggest that *Sa*M1PDH plays a role in the virulence of S. aureus USA300 by controlling mannitol biosynthesis for the defense against ROS produced by host immune cells and thus that S. aureus USA300 can gain survival advantages in the host.

We further explored these observations in a mouse infection model. Mice were infected with 2 × 10^7^ CFU of the WT strain or the knockout strains via retro-orbital injection, and either phosphate-buffered saline (PBS) or mannitol was injected into mice every 12 h postinfection for 8 days. Mice infected with WT S. aureus USA300 and subsequent injected with either PBS or mannitol died at average rates of 30% and 50%, respectively (Fig. 3C). In contrast, none of the mice that were infected with the knockout strain died after either PBS or mannitol treatment (Fig. 3C). These results implied that *Sa*M1PDH is important for bacterial pathogenicity. Unexpectedly, although the difference did not reach statistical significance, the administration of mannitol increased the death rate of mice infected with the WT strain from 30% to 50% (Fig. 3C), indicating that the presence of mannitol could also affect staphylococcal pathogenesis. However, the effect of mannitol in the mutant strain could not be evaluated by this infection model since the death rate was zero under both sets of conditions (Fig. 3C). Subsequently, the differences in the rates of bacterial colonization of WT and knockout strains in liver and kidney were compared (Fig. 3D). The colonization rates of the WT and knockout strains were quantified by the CFU assay. The survival rates of the knockout strain in liver were reduced about 10^3^-fold and 10^4^-fold under mannitol-deficient and mannitol supplementation conditions, respectively (Fig. 3D). Consistently, the survival rate of the knockout strain in kidney under the mannitol supplementation was reduced significantly (about 15-fold) compared to that of the WT strain, which showed no change under the mannitol-deficient conditions (Fig. 3D). These results suggest that the mannitol metabolism affected staphylococcal pathogenesis; wherein M1PDH plays a key role in mannitol homeostasis and plausibly acts as a molecular switch between the normal bacterial physiology and pathophysiology. Taking the results together, we propose *Sa*M1PDH as a new target for developing the next generation of antibacterial therapeutics with two parallel attributes: (i) an antivirulence approach involving inhibition of *Sa*M1PDH to accelerate the efficiency of ROS-mediated killing by host cells and (ii) an antibacterial approach involving inhibition of *Sa*M1PDH to weaken the cell wall through an imbalance in mannitol metabolism and consequent cytolysis.

### A small-molecule inhibitor of *Sa*M1PDH reduced the infection potential of S. aureus USA300.

**FIG 4:**
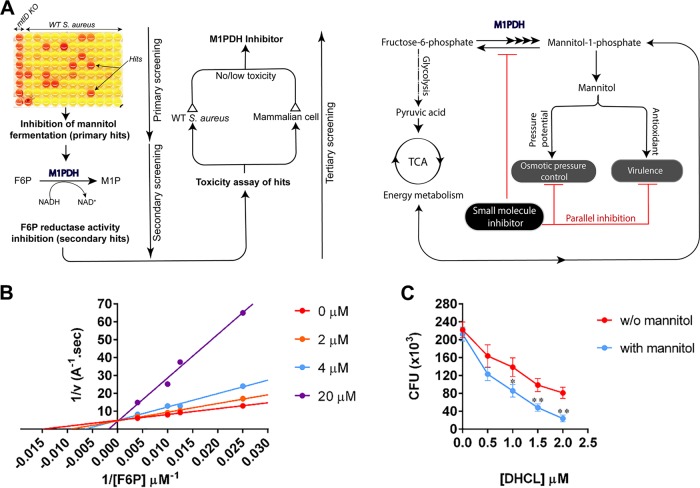
Identification of dihydrocelastrol (DHCL) as a potent *Sa*M1PDH inhibitor. (A). Schematic diagrams illustrating (left) a screening strategy to identify small-molecule inhibitors of *Sa*M1PDH and (right) the expected mode of action of identified *Sa*M1PDH inhibitors in S. aureus USA300 pathophysiology. (B) Competitive inhibitory effect of DHCL on F6P reductase activity of *Sa*M1PDH. Initial rates of *Sa*M1PDH F6P reduction reactions at various DHCL concentrations (0 to 20 μM) were calculated by measuring changes in absorbance of NADH at 340 nm. A double reciprocal Lineweaver-Burk plot of initial reaction rates versus F6P concentrations was constructed at each DHCL concentration for calculating the inhibition kinetic parameters. Dots represent data points averaged from three independent measurements with standard deviations of less than 5%. Solid lines represent linear regression curve fitting using GraphPad Prism software. TCA cycle, tricarboxylic acid cycle. (C) Synergistic effect of DHCL and mannitol on S. aureus USA300 survival rates in macrophages. Following bacterial infection and removal of extracellular bacteria by gentamicin treatment, infected RAW 264.7 cells were cultured in either control media (red) or media containing 2.75 mM mannitol (blue). To examine the effect of *Sa*M1PDH inhibitor, both culture media were supplemented with 0 to 20 μM DHCL. CFU levels of internalized bacteria were then determined by plating infected RAW 264.7 cell lysates onto the BHI agar and plotted against DHCL concentrations on a graph. Data were obtained from three independent infection experiments and are presented as means (dots) ± standard deviations of the means (whiskers). Solid lines connect data points. Statistical significance was calculated by Student's *t* test (*, *P < *0.05; **, *P < *0.01; ***, *P < *0.001).

To test the feasibility of developing small-molecule inhibitors targeting *Sa*M1PDH, a preliminary screening of the chemical libraries was performed using mannitol fermentation assays in mannitol phenol red broth media (Fig. 4A; see also Fig. S5A). The primary hits inhibiting mannitol fermentation were further validated in a secondary screening using a F6P reductase activity assay (Fig. S5B), by which we identified dihydrocelastrol (DHCL) as a potent inhibitor of *Sa*M1PDH. Kinetic inhibitory studies using various F6P and DHCL concentrations confirmed that DHCL was a competitive inhibitor of *Sa*M1PDH with a *K_i_* of 4.0 μM (Fig. 4B; see also Table 1), indicating that DHCL bound to the active site in direct competition with substrates. Next, the effect of DHCL on the virulence of S. aureus USA300 was investigated using the murine macrophage infection model. Infected RAW 264.7 cells were cultured in media with or without 2.75 mM mannitol and treated with increasing concentrations (0 to 2.0 μM) of DHCL or with dimethyl sulfoxide (DMSO) as a control. The concentration range of DHCL was determined on the basis of the concentration of DHCL that should not affect the growth of either the bacterial cells or the host cells (noninhibitory concentration [NIC]; see Fig. S5C). Even in the absence of mannitol, DHCL treatment reduced the number of surviving intracellular bacteria in a concentration-dependent manner. Moreover, in the presence of mannitol, the effect of 2.0 μM DHCL on S. aureus USA300 elimination was augmented by approximately 3-fold (Fig. 4C). From these results, we demonstrated that a small-molecule inhibitor of *Sa*M1PDH was able to exert similar effects with respect to the knockout of *mtlD* and, subsequently, that the identified inhibitor, DHCL, was capable of use as an antimicrobial drug targeting *Sa*M1PDH.

**TABLE 1:** Effects of DHCL on enzymatic kinetic parameters of *Sa*M1PDH F6P reduction activity^*a*^

DHCL concn (μM)	*K_m_*_,app_^*b*^ (μM)	*V*_max_ (A/s)	*K_m_*_,app_/*K_m_*^*c*^
0	88.6	0.22	1.00
2	104.7	0.21	1.18
4	146.5	0.19	1.65
20	516.8	0.21	5.83

a DHCL, dihydrocelastrol.

b *K_m_*_,app_, apparent *K_m_*.

c *K_m_*, substrate concentration that yields half-maximal velocity in the absence of inhibitor.

10.1128/mBio.02660-18.6 FIG S5 Functional assays for screening and identifying dihydrocelastrol (DHCL) as a potent inhibitor of *Sa*M1PDH and its effects on proliferation of S. aureus USA300 and RAW 264.7 cells. Download FIG S5, PDF file, 0.2 MB. Copyright © 2019 Nguyen et al. 2019 Nguyen et al. This content is distributed under the terms of the Creative Commons Attribution 4.0 International license.

### Molecular mechanism of stress modulation by *Sa*M1PDH.

In S. aureus, the interconversion between M1P and F6P is performed by M1PDH. Therefore, the steady states of the enzymatic reactions are expected to be controlled by cellular physicochemical parameters such as the intracellular pH (pH_i_), salinity, or redox status of the cells as observed in physiological studies (Fig. 2; see also Fig. 3). To corroborate the physiological functions of *Sa*M1PDH at the molecular level, we performed biochemical assessments. First, the dehydrogenase and reductase activities of *Sa*M1PDH were examined under the aforementioned physiological conditions to elucidate enzyme directionality. For this purpose, we measured the kinetic parameters of the dehydrogenase and reductase activities using M1P/NAD^+^ and F6P/NADH, respectively, at pH 7 (Fig. S6A and B). The *k*_cat_ and *K_m_* values for the reduction of F6P were found to be 2,540 ± 30 s^−1^ and 88.6 ± 3.4 μM, respectively, whereas the corresponding values for the oxidation of M1P were 16.8 ± 0.6 s^−1^ and 188 ± 13 μM, respectively. These data indicated that the rate of F6P reduction was about 150-fold higher than the rate of M1P oxidation. Moreover, the apparent substrate affinity for F6P was 2-fold higher. Therefore, the *k*_cat_/*K_m_* value for F6P (28.7 μM^−1^ s^−1^) was approximately 321-fold higher than the *k*_cat_/*K_m_* value for M1P (8.94 × 10^−2 ^μM^−1^ s^−1^) at neutral pH (Fig. S6A and B). This result implies that *Sa*M1PDH works as a F6P reductase enzyme under physiological conditions, and thus the synthesis of M1P is preferred.

10.1128/mBio.02660-18.7 FIG S6 Determination of Michaelis-Menten kinetic parameters for *Sa*M1PDH oxidoreductase activity. Download FIG S6, PDF file, 0.1 MB. Copyright © 2019 Nguyen et al. 2019 Nguyen et al. This content is distributed under the terms of the Creative Commons Attribution 4.0 International license.

**FIG 5:**
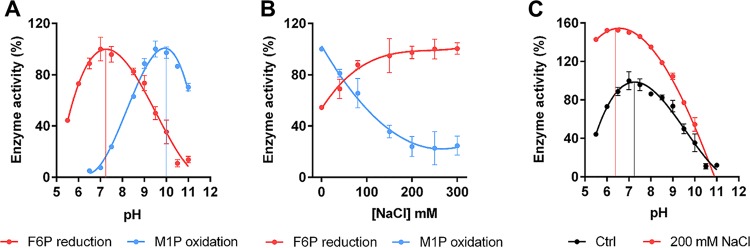
*In vitro* characterizations of *Sa*M1PDH enzymatic activities. (A) pH dependence of *Sa*M1PDH oxidoreductase activities. (B) Salt concentration dependence of *Sa*M1PDH oxidoreductase activities. In the experiments whose results are presented in panels A and B, M1P oxidation (blue) and F6P reduction activities (red) of *Sa*M1PDH were measured by monitoring changes in absorbance of NADH at 340 nm under conditions of (A) various pHs (5.0 to 11.5) and (B) various NaCl concentrations (0 to 300 mM). (C) pH dependence of *Sa*M1PDH reductase activity at 200 mM NaCl. F6P reduction activity of *Sa*M1PDH was measured by monitoring changes in absorbance of NADH at 340 nm in the presence (red) and absence (Ctrl; black) of 200 mM NaCl as a control. In panels A to C, oxidoreductase activities of *Sa*M1PDH were normalized against the maximal enzymatic capacities (indicated by thin vertical lines) measured under each set of experimental conditions and are expressed as percent activity. Each experiment was done in triplicate, and data points were presented as means (dots) ± standard deviations of the means (whiskers). Solid lines connect data points.

Second, to understand the molecular basis of the mannitol depletion in the WT strain (Fig. 2B) at higher pH, we examined the oxidoreductase activities of *Sa*M1PDH at various levels of pH (5.0 to 11.5) (Fig. 5A). Under our experimental conditions, *Sa*M1PDH reductase activity (conversion of F6P to M1P) was highest at a physiological pH (7.2), whereas dehydrogenase activity (conversion of M1P to F6P) was highest at an alkaline pH (10.0), thereby supporting our observation that the intracellular mannitol level in the WT strain at an alkaline pH was lower than that at a neutral pH (Fig. 2B). This may imply that under such stress conditions, the conversion of M1P to F6P by *Sa*M1PDH is necessary for boosting energy metabolism (glycolytic pathway), which might provide an explanation of the growth defect of the knockout strain observed at a high pH (Fig. 2A).

Third, to explain the growth defect of the knockout strain and the increased intracellular mannitol level in the WT strain under conditions of salt stress (Fig. 2A and B), the oxidoreductase activity of *Sa*M1PDH was assessed at various NaCl concentrations (0 to 300 mM) (Fig. 5B). Enzymatic assays revealed that *Sa*M1PDH F6P reduction activity increased proportionally to the NaCl concentrations and reached a plateau at 200 mM NaCl. In contrast, the M1P oxidation activity of *Sa*M1PDH decreased in a dose-dependent manner that was dependent on the NaCl concentrations and reached 20% of maximal activity at 200 mM NaCl (Fig. 5B). These results explained why the intracellular mannitol level was elevated in the WT and complemented strains but not in the knockout strain when the salt stress was imposed (Fig. 2B). Accordingly, the knockout mutant could not grow well under conditions of salt stress due to the lack of osmotic potential regulation.

In general, S. aureus strains are halotolerant and can endure a high concentration of NaCl (40). To understand the molecular mechanism of salt tolerance, we investigated the effects of salt stress on the activity of *Sa*M1PDH. It is known that salt stress is directly linked to pH stress under physiological conditions due to the activity of sodium/hydrogen exchangers in S. aureus (40, 41). In fact, sodium stress can decrease pH_i_ by the imported hydrogen ions, which occurs in response to exportation of intracellular sodium (42, 43). Accordingly, salt treatment could induce the lower pH_i_ (Fig. 1B), which actually had been observed previously in S. aureus and other Gram-positive bacteria such as Listeria monocytogenes (40, 41, 44), wherein pH_i_ was decreased upon salt treatment. Therefore, it is anticipated that under physiological conditions, salt stress not only would increase the salt concentration but also could reduce pH_i_. To test the enzymatic activity of *Sa*M1PDH under such conditions, we examined the F6P reductase activity in the presence and absence of 200 mM NaCl at various pH levels (Fig. 5C). The results revealed that F6P reductase activity was optimal at 200 mM NaCl and pH 6.5 (Fig. 5C). This may indicate that the salt endurance of S. aureus under physiological conditions was a consequence of maintaining a high mannitol level through elevated reductase activity of *Sa*M1PDH at high salt levels and under acidic pH conditions, which possibly provides a plausible explanation for one of the underlying molecular mechanisms of the high-salt endurance of S. aureus.

### Structural insight into the molecular mechanism of stress modulation by M1PDH.

**FIG 6:**
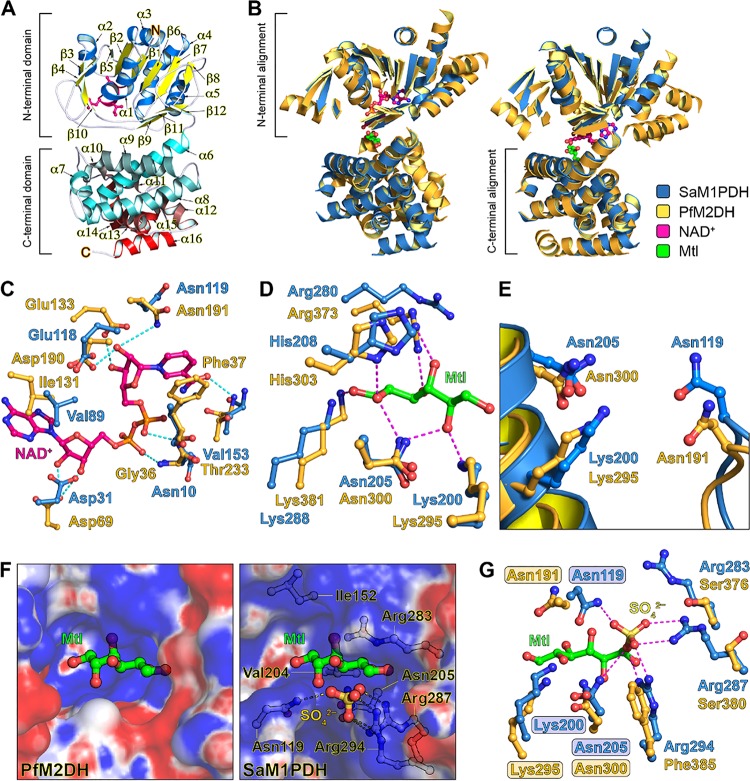
Overall structure and catalytic sites of *Sa*M1PDH. (A) Ribbon diagram of *Sa*M1PDH crystal structure. For clarity, α-helices and β-strands in the N-terminal domain are colored blue and yellow, respectively; α-helices in the C-terminal domain are colored in a gradient transition from cyan to red; and turns and loops in both domains are colored white. Consensus motif GXGXXG of the Rossmann fold in the N-terminal domain is shown in a magenta stick model. N and C termini of *Sa*M1PDH are annotated by N and C, respectively. (B) Structural comparisons of *Sa*M1PDH and *Pf*M2DH (PDB: 1M2W). Structures of *Sa*M1PDH and *Pf*M2DH are superimposed at the (left) N-terminal domains and (right) C-terminal domains. *Sa*M1PDH and *Pf*M2DH are drawn as blue and orange ribbon diagrams, respectively; for clarity, only α-helices and β-strands are shown. Mannitol (Mtl) and NAD^+^ molecules bound to *Pf*M2DH are drawn as green and magenta ball-and-stick models, respectively. This color scheme is used in the remaining panels of this figure. (C to E) Structural comparisons of *Sa*M1PDH and *Pf*M2DH at the (C) NAD^+^-binding site, (D) mannitol-binding site, and (E) catalytic triad. In panels C and D, to predict residues of *Sa*M1PDH involved in NAD^+^ and mannitol bindings, structures of *Sa*M1PDH and *Pf*M2DH are superimposed at the (C) N-terminal domains and (D) C-terminal domains as shown in panel B. Dashed lines represent hydrogen bonds between atoms in NAD^+^/mannitol and their interacting atoms in *Pf*M2DH. In the experiment whose results are presented in panel E, to predict the catalytic triad of *Sa*M1PDH, N-terminal and C-terminal domains of *Pf*M2DH were separated from each other and respectively superimposed on the intact structure of *Sa*M1PDH. (F) Electrostatic potential surfaces (contouring level of ± 5 kTe^−1^) of substrate-binding sites of (left) *Pf*M2DH and (right) *Sa*M1PDH. Structures of *Sa*M1PDH and *Pf*M2DH are superimposed at their C-terminal domains as shown in panel B. Electrostatic properties (positive charge, blue; neutral, white; negative, red) were calculated using APBS (68). The mannitol molecule was intentionally modeled in *Sa*M1PDH structure and is used as a reference for the same view to represent substrate-binding pockets of *Sa*M1PDH and *Pf*M2DH. Sulfate (yellow) and sulfate-interacting residues (white) are drawn in ball-and-stick models. (G) Structural superimposition of the active sites of *Sa*M1PDH and *Pf*M2DH. Structures of *Sa*M1PDH and *Pf*M2DH are superimposed shown in panel E. Catalytic triads of *Sa*M1PDH and *Pf*M2DH are highlighted. Magenta dashed lines represent bonds between SO_4_^2–^ (yellow ball-and-stick representation) and its interacting atoms in *Sa*M1PDH. Figures were prepared using PyMOL (http://www.pymol.org).

To understand the molecular mechanism of *Sa*M1PDH at the atomic level, we solved its crystal structure by the multiwavelength anomalous diffraction method. The final structure model was refined to a 1.7-Å resolution with *R*_work_ and R_free_ factors of 16.6% and 19.2%, respectively (Table 2). *Sa*M1PDH consists of two domains: (i) an N-terminal domain (residues 1 to 190) containing 12 β-strands (β1 to β12) and five α-helices (α1 to α5) and (ii) a C-terminal domain (residues 191 to 368) containing 11 α-helices (α6 to α16) (Fig. 6A; see also Fig. S7A). In the N-terminal domain, a six-stranded parallel β-sheet (β5-β2-β1-β6-β7-β8) forms a typical Rossmann fold, which is commonly found in nucleotide-binding proteins (45). This β-sheet is extended into two mixed β-sheets (β10-β11-β12 and β9-β3-β4), which form a barrel surrounding helix α1. The Gly-rich motif GXGXXG (residues 7 to 12), which is responsible for binding to the phosphate backbones of NAD^+^ and NADH (46, 47), is located in the loop between strand β1 and helix α1 (Fig. 6A).

**TABLE 2:** Data collection and refinement statistics

Parameter	SeMet-*Sa*M1PDH result^*a*^			
	Median	Peak	Inflection	Remote
Data collection				
Space group	*P2_1_2_1_2_1_*	*P2_1_2_1_2_1_*	*P2_1_2_1_2_1_*	*P2_1_2_1_2_1_*
Wavelength (Å)	1.0000	0.9789	0.9792	0.9640
Unit cell				
* A* (Å)	56.7	56.7		
* B* (Å)	58.1	58.1		
* C* (Å)	126.8	126.8		
α = β = γ (°)	90	90		
Resolution range (Å)	50–1.7	50–2.7	50–2.7	50–2.7
Multiplicity	6.8 (6.1)	6.8 (6.7)	6.8 (6.7)	6.8 (6.7)
Completeness (%)	98.8 (95.5)	100 (100)	100 (100)	100 (100)
<I/σ(I)>	32.7 (3.2)	31.8 (6.1)	31.2 (5.1)	31.2 (5.1)
* R*_merge_^*b*^ (%)	7.4 (42.8)	7.9 (38.5)	7.9 (39.9)	8.2 (41.1)

Refinement				
Resolution (Å)	29.27–1.70			
No. of reflections used (working/free)	44,180/1,989			
* R*_work_/*R*_free_^*c*^ (%)	16.55/19.15			
No. of atoms				
Protein	3,053			
Sulfate ion	15			
Water	456			
Average *B*-factor (Å^2^)	24.0			
No. of protein molecules in asymmetric unit	1			
Root mean square deviation from ideal geometry (71)				
Bond length (Å)	0.004			
Bond angle (°)	0.644			
Ramachandran plot (67) (%)				
Favored	97.40			
Allowed	2.60			
Outliers	0			
Clashscore	1.81			

a Values in parentheses refer to the highest resolution shell. Clashscore, the number of all-atom steric clashes per 1,000 atoms.

b *R*_merge_ = Σ_hkl_ Σ*_i_* |*I_i_*(hkl) − <*I*(hkl)>|/Σ_hkl_ Σ*_i_* I*_i_*(hkl), where *I_i_*(hkl) is the observed intensity and <*I*(hkl)> is the average intensity for multiple measurements.

c *R*_work_ = Σǁ*F*_(obs)_| − |*F*_(calc)_ǁ/Σ|*F*_(obs)_|; *R*_free_ was calculated as described for *R*_work_, but the calculation was performed for 4.31% of the total reflections that were randomly selected and omitted from refinement. calc, calculated; obs, observed.

10.1128/mBio.02660-18.8 FIG S7 Topology diagram of *Sa*M1PDH, its structural comparison with Shigella flexneri (*Sf*M1PDH); and multiple sequence alignment of *Sa*M1PDH with its homologs in other bacterial species. Download FIG S7, PDF file, 0.6 MB. Copyright © 2019 Nguyen et al. 2019 Nguyen et al. This content is distributed under the terms of the Creative Commons Attribution 4.0 International license.

To explore the biochemical implications of the presence of *Sa*M1PDH, we then compared its structure to that of P. fluorescens M2DH (*Pf*M2DH; PDB: 1M2W) in a complex with its cofactor (NAD^+^) and substrate (mannitol) (24). Individual domains were found to be well superimposed with the root mean square deviation (RMSD) values of 2.3 Å over the N-terminal 184 Cα atoms and 1.9 Å over the C-terminal 166 Cα atoms (Fig. 6B), indicating that the overall folds of the N-terminal and C-terminal domains are highly conserved between the two structures. However, superimposition of the two intact structures resulted in an RMSD value of 3.2 Å over 326 Cα atoms, indicating that the relative orientations of the N-terminal and C-terminal domains are slightly different between the two structures (Fig. 6B). This might be because of the absence of the cofactor and substrate in the structure of *Sa*M1PDH.

The structural similarity suggests that *Sa*M1PDH and *Pf*M2DH share similarities in the cofactor and substrate recognition regions. By means of comparison with the structure of *Pf*M2DH, we were able to localize the active site of *Sa*M1PDH within an interdomain pocket where the cofactor (NAD^+^/NADH) and substrate (M1P/F6P) occupy the N-terminal and C-terminal domains, respectively (Fig. 6C and D). We also identified the key residues in the active site of *Sa*M1PDH, most of which are highly conserved in *Pf*M2DH, as follows: the NAD^+^-binding residues in *Pf*M2DH (Gly36, Phe37, Asp69, Ile131, Asp190, Asn191, and Thr233) overlapped well those in *Sa*M1PDH (Asn10, Ile11, Asp31, Val89, Glu118, Asn119, and Val153) (Fig. 6C), and the mannitol-binding residues in *Pf*M2DH (Lys295, Asn300, His303, Arg373, and Lys381) superimposed well on those in *Sa*M1PDH (Lys200, Asn205, His208, Arg280, and Lys288) (Fig. 6D). Accordingly, the catalytic triad in *Pf*M2DH (Asn191, Lys295, and Asn300) is structurally identical to that in *Sa*M1PDH (Asn119, Lys200, and Asn205) (Fig. 6E), suggesting conservation in the molecular catalytic mechanisms of *Pf*M2DH and *Sa*M1PDH.

Considering the reported role of each of the catalytic residues in *Pf*M2DH (23, 24), we assumed that the active site residue Lys200 in *Sa*M1PDH plays the role of a catalytic base whereas active site residues Asn119 and Asn205 in *Sa*M1PDH are the two oxyanion hole residues. Depending on the protonation state of the Lys200 side chain, it can donate a proton in F6P reduction at a slightly acidic pH or accept a proton in M1P oxidation at an alkaline pH, thereby supporting our observation that *in vitro* oxidoreductase activity of *Sa*M1PDH is pH dependent (Fig. 5).

In addition to the pH-dependent oxidoreductase activity, we also observed salt-dependent activity of *Sa*M1PDH (Fig. 5). However, to the best of our knowledge, no study has examined the activity of *Pf*M2DH under conditions of salt regulation. Comparing the electrostatic potential surfaces of the active sites of *Sa*M1PDH to those of *Pf*M2DH, we noticed that the active site cavity of *Sa*M1PDH consists of a larger number of positively charged residues (Lys200, Arg268, Arg283, Arg287, Lys288, and Arg294) than that of *Pf*M2DH (Fig. 6F and G). In *Sa*M1PDH, hydrophobic surfaces are contributed only by Ile152 and Val204. Due to the differential levels of hydrophobicity of M1P and F6P, we assumed that at a low salt concentration, a condition which increases electrostatic forces, *Sa*M1PDH prefers M1P binding. On the other hand, at a high salt concentration, a condition which favors hydrophobic interactions, *Sa*M1PDH prefers F6P binding. This assumption is consistent with and supports our observation of the salt-dependent *in vitro* oxidoreductase activity of *Sa*M1PDH (Fig. 5B and C).

### Substrate and inhibitor binding mode of *Sa*M1PDH.

**FIG 7:**
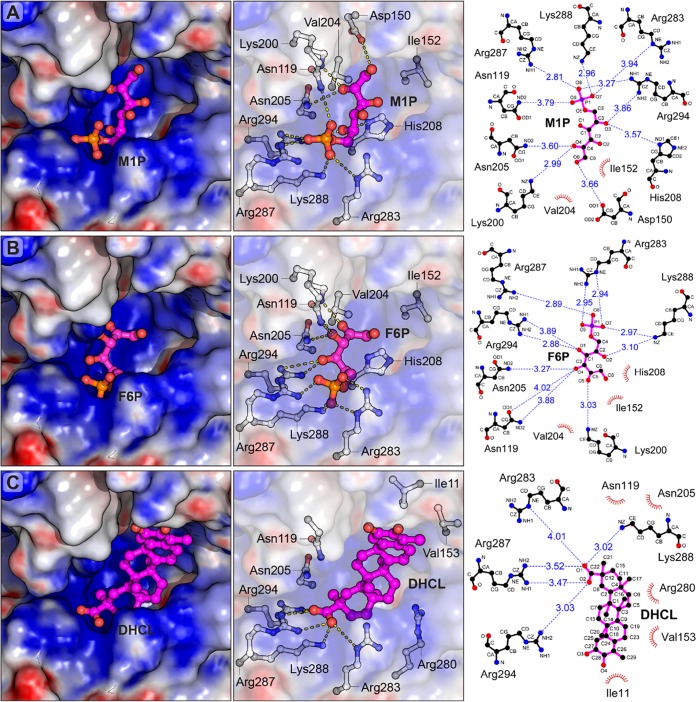
Structural docking models of *Sa*M1PDH in complexes with (A) mannitol-1-phosphate, (B) fructose-6-phosphate, and (C) dihydrocelastrol. Mannitol-1-phosphate (M1P), fructose-6-phosphate (F6P), and dihydrocelastrol (DHCL) were docked into the ligand-binding pocket of *Sa*M1PDH using UCSF Chimera (69), and only the docked chemical-*Sa*M1PDH complexes with the best binding energy scores were selected for analyses. (Left panels) Electrostatic potential surface (contouring level of ± 8 kTe^−1^) at the ligand-binding pocket. Electrostatic properties (positive charge, blue; neutral, white; negative, red) were calculated using APBS (68). Structures of *Sa*M1PDH and docked chemicals are shown as surface representations and magenta ball-and-stick models, respectively. (Middle panels) Predicted important residues of *Sa*M1PDH involved in interactions with the docked chemicals. Chemicals and *Sa*M1PDH residues are represented as magenta and white ball-and-stick models, respectively. Hydrogen and ionic bonds are represented by yellow dashed lines between interacting atoms. For clarity, the ligand-binding pocket of *Sa*M1PDH is shown as transparent electrostatic potential surfaces. (Right panels) Two-dimensional schematic diagram illustrating interactions of *Sa*M1PDH and docked chemicals. Chemicals and *Sa*M1PDH residues predicted to interact with the docked chemicals are represented as magenta and black ball-and-stick diagrams, respectively (carbon atom, black; oxygen, red; nitrogen, blue; phosphorus, purple). Hydrogen and ionic bonds are visualized as blue dashed lines between interacting atoms, and bond distances are indicated in angstrom (Å) values. Residues involved in hydrophobic interactions with the docked chemicals are visualized as brown arcs with spokes toward the contacting atoms in the chemicals. Figures were prepared by using PyMOL (http://www.pymol.org) and LigPlot^+^ v.2.1 (70).

**FIG 8:**
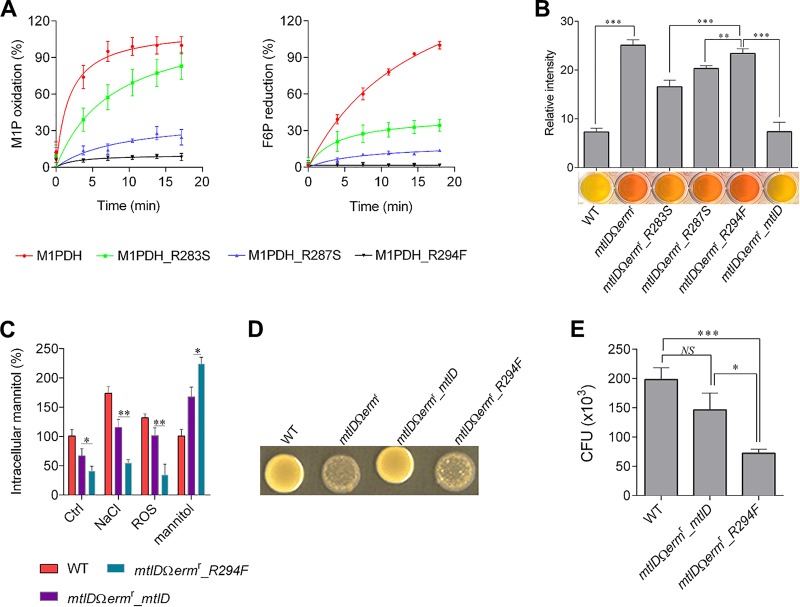
Mutational analysis of the *Sa*M1PDH phosphate-binding site. (A) *In vitro* enzymatic activities of *Sa*M1PDH mutants. Catalytic activities (left, M1P dehydrogenase; right, F6P reductase) of WT and mutant *Sa*M1PDH were examined by measuring changes in absorbance of NADH at 340 nm in a time-dependent manner. Oxidoreductase activities were normalized against the maximal enzymatic activity of WT *Sa*M1PDH and expressed as percent activities. (B) Mannitol-metabolizing activities of WT and mutant S. aureus USA300 strains. The indicated bacterial strains were cultured in the mannitol salt broth containing phenol red as a pH indicator in a 96-well plate. The plate was photographed after overnight incubation at 37°C. Failure to change broth to yellow indicates a negative mannitol fermentation result. For clarity, the relative levels of red coloring of the culture broth were quantified by densitometric analysis and plotted against S. aureus strains on a graph (shown above the photographs of culture wells). (C) Effects of various stress conditions on intracellular mannitol levels in S. aureus USA300 strains. The indicated bacterial strains were cultured in either BHI media (control [Ctrl]) or modified BHI media mimicking stress conditions (200 mM NaCl, ROS, and 27.5 mM mannitol). Intracellular mannitol was then extracted and quantified, and the levels were calculated as percentages of mannitol in S. aureus USA300 strains grown under various conditions compared to that of the WT strain grown in control media. (D) Effect of mannitol on colony morphologies of WT and mutant S. aureus USA300 strains. The indicated bacterial strains were spotted onto the BHI agar containing 27.5 mM mannitol. Plates were photographed after incubation at 37°C for 48 h. (E) Effect of mannitol on susceptibilities of WT and mutant S. aureus USA300 strains in macrophage infection assays. After infections by WT, *mtlD*Ω*erm*^r^*_mtlD*, and *mtlD*Ω*erm*^r^*_R294F* S. aureus USA300 strains followed by removal of extracellular bacteria, infected RAW 264.7 cells were cultured in media supplemented with 2.75 mM mannitol for 6 h. CFU levels of internalized bacteria were then determined by plating infected RAW 264.7 cell lysates onto BHI agar. All experiments were done in at least three independent replicates, and data were presented as means ± standard deviations of the means. Statistical significance was calculated by Student's *t* test (*, *P < *0.05; **, *P < *0.01; ***, *P < *0.001; *NS*, not significant).

While *Pf*M2DH recognizes mannitol and fructose as the substrates, *Sa*M1PDH specifically recognizes the phosphorylated forms (M1P and F6P). Thus, the active site of *Sa*M1PDH is likely to be different from that of *Pf*M2DH. Accordingly, a structural comparison revealed that the substrate-binding cavity of *Sa*M1PDH is much wider than that of *Pf*M2DH (Fig. 6F). In addition, a putative phosphate-binding pocket that was surrounded by three basic residues (Arg283, Arg287, and Arg294) was identified in the *Sa*M1PDH structure. High electron density in the pocket was modeled by SO_4_^2-^, since a high concentration of ammonium sulfate was used for the crystallization of *Sa*M1PDH (Fig. 6G). The idea of the existence of the phosphate-binding pocket is supported by the fact that the three Arg residues in the pocket are not conserved in M2DHs across bacteria but are strictly conserved among M1PDHs (Fig. 6G; see also Fig. S7B to E). Moreover, this pocket also exists in the crystal structure of M1PDH from Shigella flexneri (*Sf*M1PDH; PDB: 3H2Z), which is superimposed well on the *Sa*M1PDH structure with an RMSD value of 1.46 Å over 352 Cα atoms (Fig. S7B to D). In *Sf*M1PDH, the three conserved Arg residues stabilize a phosphate ion (Fig. S7B to D). Therefore, Arg283, Arg287, and Arg294 in *Sa*M1PDH are likely to form a structural motif that recognizes the phosphate moiety of M1P/F6P when these substrates bind to M1PDH (Fig. 6G). Supporting that conjecture, the study of docking of M1P and F6P to *Sa*M1PDH revealed that Arg287 and Arg294 play the key roles in binding the phosphate moieties of M1P and F6P whereas Arg283 makes a lesser contribution because it is further from the phosphate moiety than Arg287 and Arg294 (Fig. 6G; see also Fig. 7A and B). In addition, a molecular docking analysis of the binding of the DHCL inhibitor to *Sa*M1PDH showed that DHCL likely binds to the active site in direct competition with substrates at the phosphate-binding site (Fig. 7C), thereby inhibiting the substrate binding activity and reducing the *Sa*M1PDH activity. This result is consistent with the competitive inhibition kinetics of DHCL on *Sa*M1PDH (Fig. 4B). To confirm the structural interpretation presented above, we generated mutants of *Sa*M1PDH (R283S, R287S, and R294F), and examined their *in vitro* enzymatic activities. The catalytic activities of R287S and R294F mutants against both substrates were significantly lower than that of the WT *Sa*M1PDH. This indicates the indispensability of these two basic residues for *Sa*M1PDH activities and particularly in substrate recognition (Fig. 8A). In contrast, the catalytic activity of the R283S mutant was moderately lower than that of the WT enzyme (Fig. 8A). Therefore, the enzymatic activities of the mutants are consistent with the predicted function of the three conserved Arg residues in the phosphate binding motif.

To further validate the roles of these Arg residues *in vivo*, we complemented the *mtlD*Ω*erm*^r^ knockout strain with mutants of *mtlD* (strains *mtlD*Ω*erm*^r^_*R283S*, *mtlD*Ω*erm*^r^_*R287S*, and *mtlD*Ω*erm*^r^_*R294F*) and examined their mannitol-metabolizing activities by analysis of the corresponding color changes in mannitol phenol red broth (Fig. 8B). Consistent with the molecular analysis of enzymatic activities, strains *mtlD*Ω*erm*^r^_*R287S* and *mtlD*Ω*erm*^r^_*R294F* showed dramatically reduced and completely lost mannitol-metabolizing activities, respectively; whereas strain *mtlD*Ω*erm*^r^_*R283S* showed slightly reduced activity compared to the WT strain or the *mtlD*Ω*erm*^r^_*mtlD* strain (Fig. 8B). The physiological role of M1PDH was further analyzed using the inactive mutant complementation strain *mtlD*Ω*erm*^r^_*R294F* under conditions of salt, ROS, and mannitol stresses (Fig. 8C). This experiment confirmed that strain *mtlD*Ω*erm*^r^_*R294F* showed responses that were nearly identical to those shown by the knockout strain (Fig. 2B; see also Fig. 8C). In addition, the cell swelling and cytolysis phenotypes of the *mtlD*Ω*erm*^r^_*R294F* strain were also comparable to those of the knockout strain (Fig. 8D; see also Fig. S3B and C). Consistently, the survival rate of the *mtlD*Ω*erm*^r^_*R294F* strain was similar to that of the knockout strain and was significantly reduced compared to that of the WT strain in a RAW 264.7 macrophage infection model in the presence of mannitol (Fig. 8E). Taken together, these results strongly support the idea that Arg287 and, particularly, Arg294 are important residues in substrate recognition and thereby in the enzymatic activity of *Sa*M1PDH, which is essential for mannitol metabolism, cell wall integrity, and stress responses.

## DISCUSSION

NAD^+^/NADH-dependent oxidoreductases, M1PDH enzymes were previously found to be involved in the interconversion of M1P to F6P in the mannitol pathway (21, 48). However, very little is known about M1PDH owing to the lack of its structural and functional characterization. In this study, by comparing the *mtlD* knockout strain with WT S. aureus USA300 in terms of mannitol catabolism ability and its correlation with salinity, alkalinity, and ROS susceptibility (Fig. 2), we identified *Sa*M1PDH as an indispensable stress-alleviating enzyme, which supports the idea of the halophilic nature of S. aureus strains (49). The pH and ROS stress responses were shown to be essential for pathogen acclimation under pathophysiological conditions in the host cell (50). The elevated levels of the intracellular mannitol pool under these stress conditions provide a meaningful connection to understand how *Sa*M1PDH and mannitol play a role in protecting bacteria from these stresses in the host (Fig. 2; see also Fig. 3).

After establishing the indispensability of *Sa*M1PDH in protection against salt/pH and ROS in S. aureus, we proposed *Sa*M1PDH as a critical potential target for antibacterial therapeutics and substantiated the proposal by providing several lines of evidence. First, we described the basis of a new bactericidal strategy for S. aureus by mannitol-derived cytolysis in the absence/inhibition of *Sa*M1PDH activity. The presence of extracellular mannitol led to mannitol uptake through a PTS as reported earlier (21, 51, 52) and subsequently resulted in its accumulation due to the absence of *Sa*M1PDH activity under *mtlD* knockout conditions (Fig. 2B). Moreover, the lack of *Sa*M1PDH activity was observed to induce the expression the PTS (see Fig. S2B in the supplemental material), which further enhanced the intracellular mannitol pool, causing the inflow of excess water, cell swelling, and cytolysis (Fig. 2). It is noteworthy that mannitol or the factors (high salt, glucose, etc.) that could enhance the intracellular mannitol pool have been shown to potentiate antibiotics (β-lactam, aminoglycosides), leading to susceptibility of Escherichia coli (53), Pseudomonas aeruginosa biofilms (54), and S. aureus persisters (22). Mannitol has previously been shown to potentiate antibiotics by generating a proton-motive force (PMF) (22, 50). Therefore, mannitol metabolism seems to play an essential role in antibiotics potentiation by modulating the electrochemical gradient. However, the use of antibiotics even at lower concentrations could induce the antibiotic resistance (55). This study demonstrated an antibiotics-free alternative antibacterial approach wherein inhibition of the *Sa*M1PDH activity of mannitol metabolism followed by mannitol treatment induces the cytolysis of S. aureus cells.

Second, we demonstrated the higher susceptibility of the *mtlD* knockout strain and the effect of mannitol both in an *in vitro* murine macrophage infection model and in an *in vivo* murine infection model. The enhanced susceptibility of the S. aureus strain lacking *Sa*M1PDH activity in the *mtlD* knockout strain is presumably caused by (i) imbalance in mannitol homeostasis due to the absence/inhibition of *Sa*M1PDH activity resulting in the reduced ROS resistance potential of S. aureus and (ii) accumulation of mannitol resulting in inflow of water and cytolysis of S. aureus inside the host. (Fig. 3). Therefore, it is conceivable that a S. aureus strain devoid of *Sa*M1PDH activity due to either the absence of the *mtlD* gene or its inhibition would be extremely susceptible to host-imposed stresses, especially upon mannitol treatment.

Finally, we have shown that our proposed strategy could be implemented by a small-molecule inhibitor of *Sa*M1PDH. We exemplified the strategy by using a novel competitive inhibitor of *Sa*M1PDH, DHCL (Fig. 4; see also Fig. 7), which synergistically decreased the survival of S. aureus with mannitol treatment in an *in vitro* macrophage infection model (Fig. 4).

Mannitol was identified as one of the dominant compatible solutes in a wide spectrum of Pseudomonas putida strains and in Gluconobacter oxydans and is considered to play a role in osmoprotection during high-salt stress (10, 12). However, the molecular mechanism of stress modulation by mannitol is not well established. In this study, by investigating the molecular mechanism of directionality in *Sa*M1PDH, we established a theory of the switching activity of *Sa*M1PDH being dependent upon intracellular pH (pHi) and the salt concentration to maintain the osmotic pressure (Fig. 2B; see also Fig. 5). At lower pHi or under conditions of high salt concentrations, *Sa*M1PDH favors the biosynthesis of mannitol from fructose-6-phosphate, which causes water inflow. In contrast, at higher pHi or under conditions of low salt concentrations, *Sa*M1PDH favors the reverse direction, i.e., catabolism of mannitol, inducing water outflow (56). Interestingly, salt stress is known to decrease pHi in bacteria (40–43), indicating connections among pHi, high-salt stress, and the regulation of mannitol metabolism and homeostasis involving *Sa*M1PDH.

In the conventional mannitol metabolism cycle, two dehydrogenases (M1PDH and M2DH) are responsible for intracellular mannitol metabolism (18). However, the mannitol metabolism in bacteria seems to involve only a single dehydrogenase, i.e., either M1PDH or M2DH (see Table S1 in the supplemental material). The structure, function, and catalytic activity of M2DH have been well characterized (23–25). However, the differences in substrate recognition and specificity between these two enzymes remained unknown. In this study, our atomic resolution structure of *Sa*M1PDH (1.7 Å) and its structural comparison with that of *Pf*M2DH in complex with mannitol and NAD^+^ provided structural insights into such differences in substrate recognition and specificity (23–25). The substrate-binding site of *Sa*M1PDH is located in the interdomain interface as found in *Pf*M2DH. The *Sa*M1PDH substrate-binding cavity is larger than that of *Pf*M2DH and is highly positively charged owing to the presence of arginine residues R283, R287, and R294. These three Arg residues appear to be necessary for the recognition of the phosphate moiety as they are located at the expected position of the phosphate moiety-binding pocket and are highly conserved with respect to M1PDH among bacteria (Fig. 6; see also Fig. S7B to E). The role of the three arginine residues in the *Sa*M1PDH substrate-binding cavity was further corroborated by docking analysis and mutational validation of *Sa*M1PDH (Fig. 8). Interestingly, the docking study results also suggested that the competitive inhibitor of *Sa*M1PDH, DHCL, would also bind the phosphate moiety-binding pocket and thereby competes with the substrates to block the *Sa*M1PDH activity (Fig. 7).

10.1128/mBio.02660-18.9 TABLE S1 Presence (○) and absence (×) of M1PDH and M2DH in different S. aureus strains, plants, and fungi. Download Table S1, PDF file, 0.1 MB. Copyright © 2019 Nguyen et al. 2019 Nguyen et al. This content is distributed under the terms of the Creative Commons Attribution 4.0 International license.

In conclusion, by applying comprehensive structural, biochemical, and physiological approaches, we found that *Sa*M1PDH performs as a molecular switch between energy provision and osmotic pressure maintenance depending on external cues such as pH, salt, and redox status, which control *Sa*M1PDH activities and directionality for mannitol consumption or biosynthesis. We also suggest that the antibacterial effect of the *Sa*M1PDH inhibitor can be enhanced when combined with the presence of mannitol. To the best of our knowledge, this is the first report demonstrating that *Sa*M1PDH acts as a potent antibacterial target by the activity of its mechanism and identifying its specific inhibitor, DHCL. Indispensability, ubiquity, and uniqueness in a wide range of S. aureus strains (Table S1) make *Sa*M1PDH a potent antibacterial and an antivirulence target and thus plausibly effective in the treatment of multidrug-resistant S. aureus infections.

## MATERIALS AND METHODS

### Supplemental materials and methods.

Additional descriptions of materials and methods used in this study are presented in Text S1 in the supplemental material.

10.1128/mBio.02660-18.1 TEXT S1 Supplemental materials and methods. Download Text S1, PDF file, 0.1 MB. Copyright © 2019 Nguyen et al. 2019 Nguyen et al. This content is distributed under the terms of the Creative Commons Attribution 4.0 International license.

### Materials.

All chemicals used in this study were purchased from Sigma-Aldrich (St. Louis, MO, USA) unless specified otherwise. When necessary, bacterial growth media were supplemented with appropriate antibiotics at the following working concentrations: kanamycin 30 μg/ml and ampicillin 100 μg/ml for Escherichia coli and chloramphenicol 25 μg/ml and erythromycin 10 μg/ml for Staphylococcus aureus. The primers (Bioneer Corporation, South Korea), plasmids, and bacterial strains used in this study are listed in Table S2 in the supplemental material. The murine macrophage RAW 264.7 cell line (ATCC TIB-71) was purchased from the American Type Culture Collection (ATCC, USA) and maintained following the supplier’s instructions.

10.1128/mBio.02660-18.10 TABLE S2 Primers, plasmids, and bacterial strains used in this study. Download Table S2, PDF file, 0.2 MB. Copyright © 2019 Nguyen et al. 2019 Nguyen et al. This content is distributed under the terms of the Creative Commons Attribution 4.0 International license.

### Methods. (i) Staphylococcus aureus cell growth assays performed under abiotic stress conditions.

To generate the abiotic stresses, the brain heart infusion (BHI) media were modified as follows: (i) to mimic an alkaline condition, the BHI media were adjusted to a pH of 10.0 by addition of NaOH; (ii) to mimic a high-osmoticity condition, the BHI media were supplemented with 0.2 M NaCl. After an overnight culture in the unmodified BHI media at 37°C, the S. aureus cultures were diluted to an optical density at 600 nm (OD_600_) of 0.05 in either unmodified BHI media or modified BHI media mimicking abiotic stresses. Bacterial suspensions were then divided into aliquots (200 μl) and placed into each well of 96-well plates, and the plates were incubated at 37°C. The cell growth was monitored by measuring the OD_600_ at 1-h intervals for 16 h on an Infinite M200 Microplate Reader (Tecan, USA) or by quantifying the number of bacteria by CFU assays at 5-h intervals for 15 h.

**(ii) Determination of the intracellular mannitol levels using a colorimetric assay.** To determine the accumulations of the intracellular mannitol in S. aureus strains under conditions of various abiotic stresses, S. aureus strains were grown at 37°C for 6 h in either BHI media or modified BHI media mimicking the stress conditions as follows: (i) BHI media were adjusted to a pH of 10 by addition of NaOH; (ii) BHI media were supplemented with 0.2 M NaCl; (iii) BHI media were supplemented with ROS-generating chemicals (50 μM H_2_O_2_, 10 μM FeSO_4_, and 10 μM NaI); (iv) BHI media were supplemented with 27.5 mM d-mannitol. After two washes with phosphate-buffered saline (PBS), a 100-mg volume of bacterial cell pellets was treated with 5 units of lysostaphin at 37°C for 30 min in a 0.5-ml volume of buffer containing 40 mM Tris-HCl (pH 7.5), 3.5 mM EDTA, and 5.0 mM cysteine hydrochloride. A 1-ml volume of a phenol-chloroform (1:1) mixture was then added into the bacterial cell lysates to separate proteins, lipids, and water-insoluble metabolites from water-soluble metabolites. Because mannitol is much more soluble in water than in organic solvents, the intracellular mannitol prefers residing in the aqueous phase rather than in the organic phase. After vortex mixing was performed for 2 min, the mixtures were centrifuged at 9,300 × *g* for 10 min at room temperature, and the top aqueous layers were carefully collected. Following a passage of the collected aqueous layers through a mixture (1:1) of strong cation-exchange resins (Dowex 50WX4; hydrogen form, 100 to 200 mesh) and strong anion-exchange resins (Dowex 1X8; chloride form, 200 to 400 mesh), the eluents containing neutral sugars were recovered and subjected to a colorimetric assay of mannitol as described by Sanchez (57). Briefly, mannitol in the eluents was specifically oxidized by periodate at a pH of 3.0 in the presence of acetylacetone, ammonium acetate, and sodium thiosulfate. After heating the reaction mixtures at 100°C for 2 min, the intracellular mannitol level was determined by measuring the absorbance of the resultant yellow color at 412 nm on a model V-750 spectrophotometer (Jasco, Japan).

**(iii) Triton X-100-induced autolysis assays.** Overnight BHI cultures incubated at 37°C of S. aureus strains were diluted in the BHI media to an OD_600_ of 0.05. Bacteria (10 μl) were then spotted onto either BHI agar or BHI agar containing 27.5 mM mannitol. After incubation at 37°C for 48 h, bacterial spots were scraped from the agar surfaces and suspended in PBS. Following two washes with PBS, bacterial suspensions were diluted to an OD_600_ of 0.9 in PBS containing 0.05% Triton X-100, and 200-μl volumes of the suspensions were divided into aliquots and placed into each well of a 96-well plate. Cell lysis were examined by monitoring the OD_600_ at 2-h intervals for 12 h at room temperature on an Infinite M200 Microplate Reader (Tecan, USA) or by quantification of the surviving bacterial counts by CFU assays at intervals of 4 h for 12 h.

**(iv) S. aureus cell morphology assays using scanning electron microscope (SEM) analysis.** The WT and *Sa*M1PDH knockout (*mtlD*Ω*erm*^r^) S. aureus strains were grown at 37°C for 48 h in either BHI media or BHI media supplemented with 5 mM mannitol. After three washes with PBS, S. aureus cells were fixed with 2% glutaraldehyde–PBS for 12 h at room temperature. The cells were then gradually dehydrated in 10%, 20%, 30%, 50%, 70%, and 90% ethanol at room temperature for 5 min at each ethanol concentration and finally suspended in the absolute ethanol. After adjustment of the cell densities to an OD_600_ of 1.0 in absolute ethanol, 10-μl volumes of the cell suspensions were placed on the silicon wafer substrates and air dried. Following sputtering performed with platinum, air-dried specimens were observed on a JSM-6390A scanning electron microscope (JEOL, Japan) with an accelerating voltage of 15 kV at a working distance of 10.1 mm and a magnification of ×30,000. The relative diameters of 40 to 100 bacterial cells that were randomly selected from each S. aureus strain appearing the SEM photographs were measured using ImageJ software (58).

**(v) *Sa*M1PDH oxidoreductase activity assays.** In a typical reaction, a 13 nM concentration of either purified WT or mutant *Sa*M1PDH was incubated with a 13 μM concentration of a substrate (mannitol-1-phosphate [M1P] or fructose-6-phosphate [F6P]) and a 200 mM concentration of a corresponding cofactor (NAD^+^ or NADH) in a reaction volume of 200 μl in 96-well plates. The dehydrogenase and reductase activities of *Sa*M1PDH were determined by measuring the changes in the absorbance of NADH at 340 nm on an Infinite M200 Microplate Reader (Tecan, USA) after 10 min of incubation at room temperature unless otherwise stated.

To examine *Sa*M1PDH oxidoreductase activities in a time-dependent manner, the reactions were carried out in buffers containing 25 mM HEPES (pH 7.5). To examine the salt concentration dependences of *Sa*M1PDH oxidoreductase activities, the reactions were carried out in buffers containing 25 mM HEPES and 0 to 300 mM NaCl. To examine the pH dependences of *Sa*M1PDH oxidoreductase activities, the reactions were carried out in different buffers as follows: 25 mM MES (morpholineethanesulfonic acid) at pH 5.0 to 7.0, 25 mM HEPES at pH 7.5 to 8.5, 25 mM CHES [2-(cyclohexylamine)ethanesulfonic acid] at pH 8.5 to 10.0, and 25 mM CAPS (*N*-cyclohexyl-3-aminopropanesulfonic acid) at pH 10.0 to 11.0. These reaction buffers, supplemented with 200 mM NaCl, were used to examine the effects of both NaCl and pH on the F6P reductase activity of *Sa*M1PDH.

**(vi) Macrophage infection assays.** Prior to the S. aureus infection, the murine macrophage RAW 264.7 cells were cultured in low-glucose Dulbecco’s modified Eagle’s medium (DMEM) (Welgene, South Korea) supplemented with 10% fetal bovine serum (FBS) (Welgene, South Korea). The S. aureus cells, after being cultured overnight, were harvested by centrifugation (4,000 rpm for 5 min), followed by three washes in cold PBS. The bacterial cells were resuspended in DMEM and were then used to infect RAW 264.7 cells at a multiplicity of infection (MOI) of 100:1 (30–32) at 37°C in a CO_2_ incubator (5% CO_2_) for 30 min. To kill the extracellular S. aureus in a GPA, infected RAW 264.7 cells were treated with 100 μg/ml gentamicin for 1 h at 37°C in a CO_2_ incubator. Following two washes with DMEM, the infected RAW 264.7 cells were continuously incubated with DMEM containing 2.75 mM mannitol and 100 μg/ml gentamicin for 6 h at 37°C in a CO_2_ incubator. To examine the synergistic effect of dihydrocelastrol (DHCL) and mannitol on S. aureus strains infecting the macrophages, the culture media were further supplemented with various concentrations (0 to 20 μM) of DHCL. After three washes with PBS, infected RAW 264.7 cells were lysed in PBS containing 0.05% Triton X-100. The RAW 264.7 cell lysates were serially diluted and plated onto the BHI agar to enumerate the internalized S. aureus cells. To validate the GPA-based data, the enzyme protection assay (EPA) was performed in parallel wherein host-cell impermeable lysostaphin was used to kill extracellular S. aureus (33) (Fig. S4A).

**(vii) Murine model of systemic infection.** The WT and M1PDH knockout (*mtlD*Ω*erm*^r^) S. aureus strains were grown in tryptic soy broth (TSB) at 37°C overnight with shaking. The cell cultures were then diluted 1:100 into the fresh TSB and allowed to grow at 37°C until the cultures reached an OD_600_ of 0.8. Bacterial strains were collected by centrifugation, washed thrice, and suspended in PBS. A total of 100 μl of the bacterial cell suspension (2 × 10^7^ CFU) was administered intravenously via retro-orbital injection into each of 15 sex-matched 8-week-old C57BL/6 mice. Subsequently, the same volume of either PBS or PBS containing mannitol was injected intravenously into these mice at 12-h intervals during the course of the experiment. Mice were monitored for survival over 8 days. Subsequently, the surviving mice were subjected to organ isolation, followed by the estimation of bacterial colonization in kidney and liver by CFU assay. The experiment was carried out in strict accordance with the recommendations in the Guide for the Care and Use of Laboratory Animals of the National Institutes of Health. The survival curves were compared using a log rank (Mantel-Cox) test, and the statistical significance of results of comparisons of the levels of bacterial burdens in the indicated organs was determined using the *F*-test with GraphPad Prism 6.

**(viii) Determination of kinetic parameters for *Sa*M1PDH oxidoreductase activity.** Determinations of the Michaelis-Menten kinetic parameters of *Sa*M1PDH oxidoreductase activity followed the procedure described above for examining the time-dependent *Sa*M1PDH enzymatic activities with the following modifications. For the reductase activity, purified *Sa*M1PDH was incubated at a concentration of 0.1 ng/ml with 200 mM NADH and various concentrations (0 to 450 μM) of F6P. For the oxidase (dehydrogenase) activity, purified *Sa*M1PDH was incubated at a concentration of 30 ng/ml with 200 mM NAD^+^ and various concentrations (0 to 250 μM) of M1P. At each substrate concentration, initial enzymatic velocity was determined after a 1-min reaction at 30°C by measuring the change in the absorbance of NADH in the reaction mixture with substrate compared to that in the control reaction without substrate and expressed as absorbance change per second (A/s). Graphs were constructed by plotting the initial enzymatic velocities against the substrate concentrations. The Michaelis-Menten kinetic parameters, *K_m_* and *V*_max_, were calculated from a nonlinear regression curve-fitting analysis using GraphPad Prism 6.

To examine the effect of dihydrocelastrol (DHCL) on the Michaelis-Menten kinetic parameters of F6P reductase activity, reactions were done that were similar to those described above but that were performed in the presence of various DHCL concentrations (0 to 20 μM). After calculation of the initial enzymatic velocity at each substrate and inhibitor concentration, a double reciprocal Lineweaver-Burk plot of the initial reaction rates versus the F6P concentrations was constructed at each DHCL concentration. The apparent Michaelis-Menten kinetic parameters, $K_{m}^{\text{app}}$ and $\;V_{\text{max}}^{\text{app}}$, were calculated from a linear regression curve-fitting analysis using GraphPad Prism 6. The data are summarized in Table 1.

**(ix) Protein crystallization.** In a hanging-drop vapor diffusion setup, drops containing 1 μl of selenomethionine (SeMet)-*Sa*M1PDH at a concentration of 35 mg/ml and 1 μl of a crystallization reagent [1.8 M (NH_4_)_2_SO_4_, 0.1 M cacodylate pH 6.5, and 0.01 M CoCl_2_] were equilibrated against 0.5 ml of the reagent reservoir at 22°C. Diffraction-quality crystals of SeMet-*Sa*M1PDH were obtained after 1 month.

**(x) Data collection and structure determination.** Before exposure to the X-ray beam, crystals were soaked in the crystallization reagent containing 20% glycerol and flash-frozen in a cold nitrogen stream. All data were collected at beamline 4A of the Pohang Accelerator Laboratory (PAL; South Korea) and processed using the HKL-2000 program package (59). A multiwavelength anomalous diffraction (MAD) data set was collected from a single SeMet-*Sa*M1PDH crystal at a resolution of 2.7 Å at three wavelengths (peak, 0.9789 Å; inflection, 0.9792 Å; remote, 0.9640 Å). A 1.7-Å data set collected from another single SeMet-*Sa*M1PDH crystal at a wavelength of 1.0 Å was used for the refinement of the final model. Data collection statistics are summarized in Table 2.

The positions of four selenium atoms were determined, and an initial map was calculated from the MAD data set using the SOLVE program (60). The density modifications were carried out using the RESOLVE program (61, 62), and approximately 80% of *Sa*M1PDH residues were automatically built into the density-modified map. This incomplete model was then used as a search template for molecular replacement against the 1.7-Å data set using the MOLREP program (63). The automatic model building was then performed using ARP/wARP software (64) with the MOLREP solution as the input. The manual model building and correction were conducted using COOT software (65), followed by refinement using the PHENIX package (66). The final model consisted of all 369 amino acids (residues 1 to 368), with an *R* factor and *R*_free_ factor of 16.55% and 19.15%, respectively. The structural validation performed using the MolProbity program (67) showed that the final structure had an excellent stereochemistry with no residues lying in disallowed regions of the Ramachandran plot. The structural refinement and validation data are summarized in Table 2. All structural figures were generated using PyMOL (http://www.pymol.org).

**(xi) Mannitol fermentation assays.** Examination of the mannitol-metabolizing capacities of different S. aureus strains followed the procedure described above for the primary screening assay to identify the inhibitor hits of *Sa*M1PDH but was performed in the absence of DMSO and the natural compounds. After the plates were photographed, the levels of red coloring of the mannitol salt broths were relatively quantified for each strain using ImageJ software (58).

**(xii) Statistical analyses.** All experiments were done in at least two independent replicates. Unless otherwise mentioned, statistical significance differences between the sample groups were determined by Student's *t* test (*, *P < *0.05; **, *P < *0.01; ***, *P < *0.001; *NS*, not significant).

**(xiii) Data availability.** The coordinates and structure factor of S. aureus M1PDH have been deposited in the Protein Data Bank under accession code 5JNM.
