# Supplementary material for: Targeting Mannitol Metabolism as an Alternative Antimicrobial Strategy Based on the Structure-Function Study of Mannitol-1-Phosphate Dehydrogenase in Staphylococcus aureus
Source: mBio. 2019 Jul 9;10(4):e02660-18. doi: 10.1128/mBio.02660-18 (PMC6623548; doi:10.1128/mBio.02660-18)
Supplement: FIG S1 [file mBio.02660-18-sf001.pdf]

Fig. S1

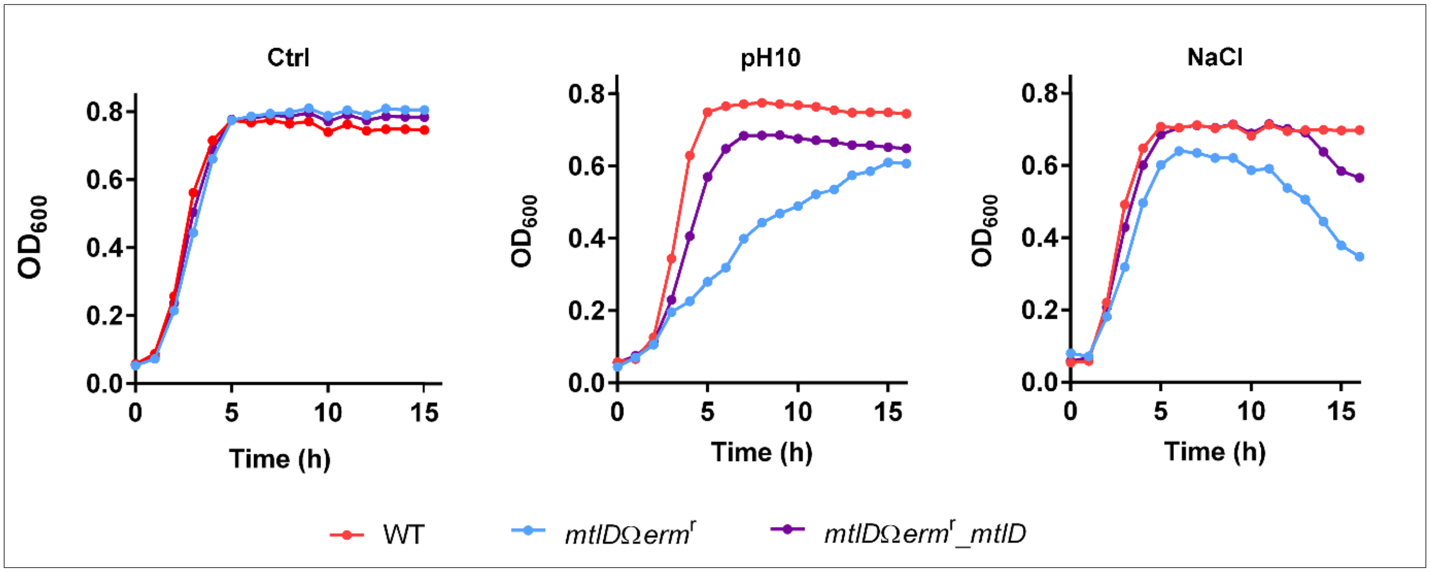

Fig. S1. Growth profiles of *S. aureus* USA300 strains under control physiological and various stress conditions. Growths of WT, knockout (*mtlD*Ω*erm*[r]), and *mtlD*Ω*erm*[r] complemented with wild type *mtlD* (*mtlD*Ω*erm*[r]*_mtlD*) *S. aureus* strains were assessed in 96-well plates by monitoring optical density at 600 nm (OD_{600}) up to 16 h at 37 °C in BHI media without (Ctrl) or with adjusted alkaline pH (pH 10) using NaOH or supplemented with 0.2 M NaCl (NaCl). Data points are averaged from three independent measurements with standard deviations less than 5%.
