# Supplementary material for: Targeting Mannitol Metabolism as an Alternative Antimicrobial Strategy Based on the Structure-Function Study of Mannitol-1-Phosphate Dehydrogenase in Staphylococcus aureus
Source: mBio. 2019 Jul 9;10(4):e02660-18. doi: 10.1128/mBio.02660-18 (PMC6623548; doi:10.1128/mBio.02660-18)
Supplement: FIG S2 [file mBio.02660-18-sf002.pdf]

Fig. S2

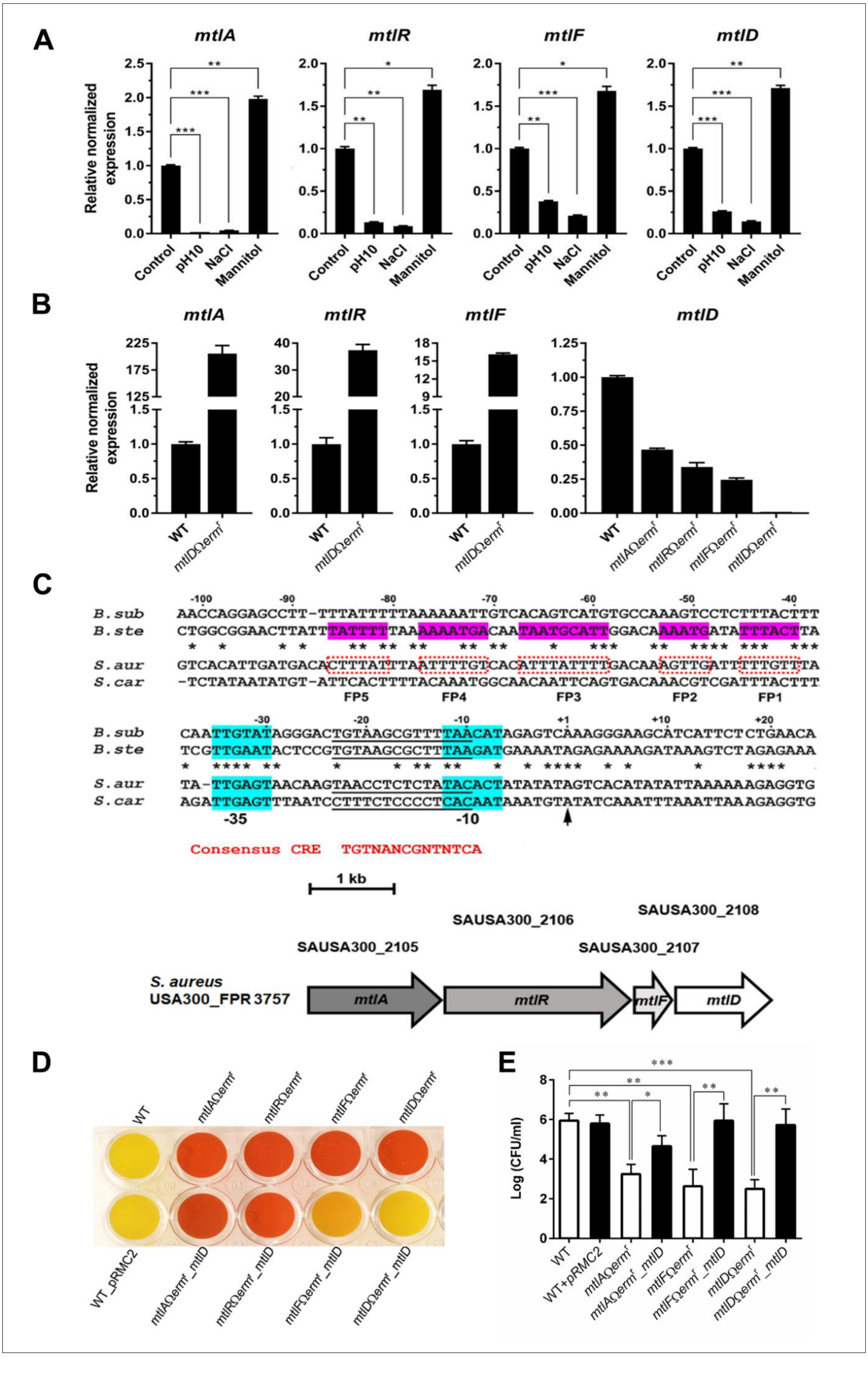
A
mtlA
mtlR
mtlF
mtlD
Relative normalized expression
Control
pH10
NaCl
Mannitol
B
mtlA
mtlR
mtlF
mtlD
Relative normalized expression
WT
mtlDΩerm^r
mtlAΩerm^r
mtlRΩerm^r
mtlFΩerm^r
C
B.sub AACCAGGAGCCTT-TTTATTTTTAAAAAAATTGTCACAGTCATGTGCCAAAGTCCTCTTTACTTT
B.ste CTGGCGGAACTTATTTATTTTTAAAAAATGACAATAATGCATTGGACAAAATGATATTTTACTTA
S.aur GTCACATTGATGACACTTTATTTAAATTTTGTCACATTTATTTTGACAAAGTTGATTTTTGTTTA
S.car -TCTATAATATGT-ATTCACTTTTACAAATGGCAACAATTCAGTGACAAACGTCGATTTACTTT
FP5
FP4
FP3
FP2
FP1
B.sub CAATTGTATAGGGACTGTAAGCGTTTTAACATAGAGTCAAAGGGAAGCATCATTCTCTGAACA
B.ste TCGTTGAATACTCCGTGTAAGCGCTTTAAGATGAAAATAGAGAAAAGATAAAGTCTAGAGAAA
S.aur TA-TTGAGTAACAAGTAACCTCTCTATACACTATATATAGTCACATATATTAAAAAAGAGGTG
S.car AGATTGAGTTTAATCCTTTCTCCCCTCACAATAAATGTATATCAAATTTAAATTAAAGAGGTG
-35
-10
Consensus CRE TGTNANCGNTNTCA
1 kb
SAUSA300_2105
SAUSA300_2106
SAUSA300_2107
SAUSA300_2108
S. aureus
USA300_FPR3757
mtlA
mtlR
mtlF
mtlD
D
WT
mtlAΩerm^r
mtlRΩerm^r
mtlFΩerm^r
mtlDΩerm^r
WT_pRMC2
mtlAΩerm^r_mtlD
mtlRΩerm^r_mtlD
mtlFΩerm^r_mtlD
mtlDΩerm^r_mtlD
E
Log (CFU/ml)
WT
WT+pRMC2
mtlAΩerm^r
mtlAΩerm^r_mtlD
mtlFΩerm^r
mtlFΩerm^r_mtlD
mtlDΩerm^r
mtlDΩerm^r_mtlD

Fig. S2. Transcriptional profiles of *mtlARFD* in wild type *S. aureus* USA300 and in isogenic mutants of *bursa aurealis* transposon in individual genes of *mtl*-operon for phenotypic assessments of mannitol catabolism and alkalinity stress. **(A)** qRT-PCR data showing the expression profile of *mtlA*, *mtlR*, *mtlF*, and *mtlD* under physiological (control); and pH, salt & mannitol stress conditions. Briefly, *S. aureus* cells were exposed to either BHI media (control) or modified BHI media mimicking stress conditions (pH 10.0, 0.2 M NaCl, and 27.5 mM mannitol) for 30 min at 37 °C. The *S. aureus* cells were harvested, and total RNA from individual samples were isolated and reversed transcribed. Relative abundance of each gene within *mtlARFD* operon was determined by a qRT-PCR analysis wherein *rpoB*, *rho* (1); and 16S rRNA genes served as internal controls. The mRNA levels of target genes were compared as control *versus* stress conditions indicated in graphs. **(B)** The expression profile of individual genes of *mtl*-operon in isogenic mutants. **(C)** Phenotypic assessment of mannitol operon mutants using mannitol catabolism assay wherein **(I)** showing the sequence similarity of the mannitol operon promoter region of *S. aureus* USA300 with *B. subtilis*, *B. stearothermophilus, S. carnosus* (2), and **(II)** depicts the arrangement of mannitol operon (*mtlARFD*) in *S. aureus* USA300 FPR3757 (NC_007793.1). **(D)** Assessment of mannitol catabolism in WT *S. aureus* or its pRMC2 supplemented strains (empty plasmid control) served as controls in mannitol catabolism assay (WT and WT_pRMC2). The isogenic insertion mutants in *mtl*-operon, *mtlARFD* (*mtlA*Ω*erm*^{r}*,* *mtlR*Ω*erm*^{r}, *mtlF*Ω*erm*^{r}, and *mtlD*Ω*erm*^{r}) were supplemented with wild type *Sa*M1PDH encoding plasmid, pRMC2_*mtlD* and were designated as *mtlA*Ω*erm*^{r}*_mtlD,* *mtlR*Ω*erm*^{r}*_mtlD,* *mtlF*Ω*erm*^{r}*_mtlD*, and *mtlD*Ω*erm*^{r}*_mtlD*. These ten strains were cultured under static culture conditions in mannitol (55 mM) phenol red broth for 24 h at 37 °C before being photographed. The phenol red color turned into yellow, which indicates mannitol catabolism. Supplementation of pRMC2_*mtlD* in *mtlA*Ω*erm*^{r} (*mtlA*Ω*erm*^{r}*_mtlD*) and *mtlR*Ω*erm*^{r} (*mtlR*Ω*erm*^{r}*_mtlD*) mutants could not recover the mannitol catabolism ability. Interestingly, the *mtlF*Ω*erm*^{r} mutant supplementation of *mtlD* (*mtlF*Ω*erm*^{r}*_mtlD*) could partially recover the mannitol catabolism compared to the complemented strain of *mtlD*Ω*erm*^{r} (*mtlD*Ω*erm*^{r}*_mtlD*). This result indicated that the *mtlA* seems to be a major component responsible for mannitol uptake in *S. aureus* USA300. **(E)** Assessment of comparative alkalinity stress response of mannitol-specific PTS mutants, *mtlA*Ω*erm*^{r} and *mtlF*Ω*erm*^{r} with *mtlD*Ω*erm*^{r} mutant. Overnight cultures of indicated *S. aureus* strains were diluted to 1×10^8 cells in the alkaline BHI media (pH 10) and incubated at 37 °C for 5 h. Bacterial counts were quantified by using CFU assays. All the *mtlA*Ω*erm*^{r}, *mtlF*Ω*erm*^{r}, and *mtlD*Ω*erm*^{r} mutants were found to be susceptible compared to the WT exposed to the same stress conditions. The comparable survival of *mtlF*Ω*erm*^{r}*_mtlD* strain with *mtlD*Ω*erm*^{r}*_mtlD* and WT or WT_pRMC2, further supported that the mannitol-specific PTS enzyme IIBC is the major component of mannitol-specific PTS system. Data are presented as means ± standard deviation, which were determined from two biological replicates. Statistical significance was calculated by Student's *t*-test (* $p < 0.05$, ** $p < 0.01$, *** $p < 0.001$).

References

1. Sihto HM, Tasara T, Stephan R, Johler S. 2014. Validation of reference genes for normalization of qPCR mRNA expression levels in *Staphylococcus aureus* exposed to osmotic and lactic acid stress conditions encountered during food production and preservation. FEMS Microbiol Lett 356:134-40.
2. Henstra SA, Tuinhof M, Duurkens RH, Robillard GT. 1999. The *Bacillus Stearothermophilus* mannitol regulator, MtlR, of the phosphotransferase system - A DNA-binding protein, regulated by HPr and IICB *mtl*-dependent phosphorylation. J Biol Chem 274:4754-4763.
