# Supplementary material for: Targeting Mannitol Metabolism as an Alternative Antimicrobial Strategy Based on the Structure-Function Study of Mannitol-1-Phosphate Dehydrogenase in Staphylococcus aureus
Source: mBio. 2019 Jul 9;10(4):e02660-18. doi: 10.1128/mBio.02660-18 (PMC6623548; doi:10.1128/mBio.02660-18)
Supplement: FIG S3 [file mBio.02660-18-sf003.pdf]

Fig. S3

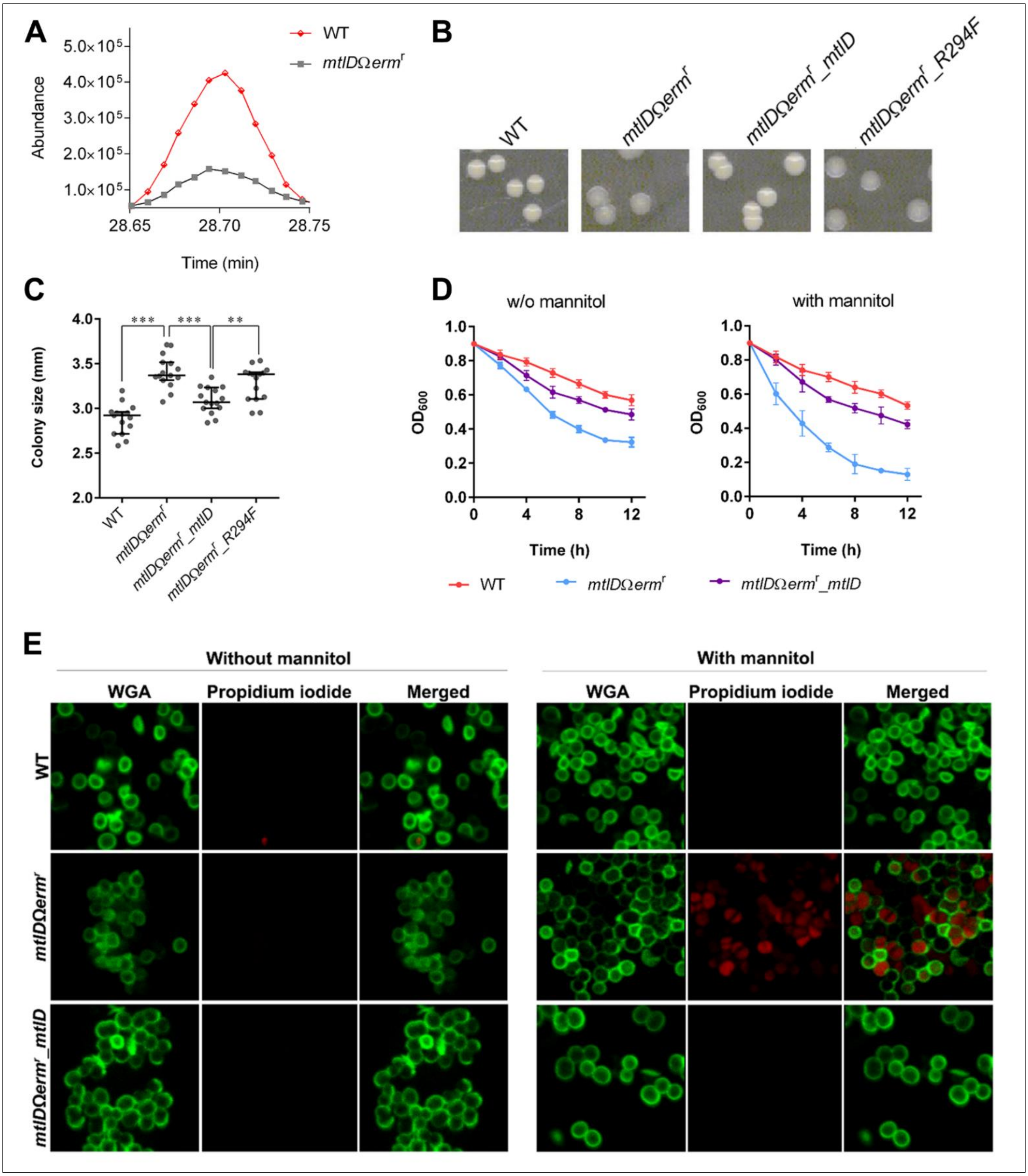

Fig. S3. Assessment of intracellular accumulations of mannitol in *S. aureus* strains and its impact on colony morphology of various *S. aureus* USA300 strains under control and stress conditions. (A) Gas chromatography-mass spectrometry (GC-MS) analysis of intracellular mannitol. Briefly, cytoplasmic contents were extracted from cell pellets of WT and *Sa*M1PDH knockout (*mtlD*Ω*erm*^{r}) *S. aureus* strains by flash-freezing cells in liquid nitrogen followed by lysostaphin treatment and sonication. After derivatization, samples were subjected to GC-MS analysis in which D-mannitol and adonitol were used as a reference and

an internal standard, respectively. After comparing spectra of chromatogram peaks with an electron impact mass spectrum library NIST08 (NIST, USA) followed by normalization, relative abundance of mannitol in each sample was determined by absorbance peak of mannitol in chromatography retention profiles. Data points (WT, diamond; knockout, square) represent absorbance of mannitol recorded at each chromatographic retention time. Solid lines (WT, red; knockout, gray) connect data points. Data are representative of at least three independent measurements. The *Sa*M1PDH knockout (*mtlD*Ω*erm*^{r}) was found to accumulate significantly higher amount of mannitol than that of WT *S. aureus* USA300. **(B)** Effect of mannitol on colony morphologies of *S. aureus* strains wherein colonies of WT, knockout (*mtlD*Ω*erm*^{r}), WT complemented (*mtlD*Ω*erm*^{r}*_mtlD*), and R294F mutant complemented (*mtlD*Ω*erm*^{r}*_R294F*) *S. aureus* strains on mannitol-containing nutrient agar. Indicated *S. aureus* strains were plated onto BHI agar containing 27.5 mM mannitol. After incubation at 37 °C for 48 h, plates were photographed. Images are representative of at least two independent experiments for each strain. Colony sizes of WT, knockout, WT complemented, and R294F mutant complemented *S. aureus* strains on mannitol-containing nutrient agar. (**C**) From the photographs,15–25 colonies from each strain were randomly selected for diameter measurements. Horizontal lines represent means of colony sizes, whereas whiskers represent one standard deviation of the means. Statistical significance was calculated by Student's *t*-test ($^{**}p < 0.01$, $^{***}p < 0.001$). **(D)** Optical density-based Triton X-100-induced autolysis assay depicting the effect of mannitol on *S. aureus* USA300 cell lysis. Briefly, after being cultured in either (left), BHI media or (right) BHI media containing 27.5 mM mannitol, bacterial cell densities were adjusted to an OD_{600} of 0.9 in PBS containing 0.05% Triton X-100 (1, 2). The time-dependent OD_{600} was monitored at room temperature for 12 h. The reduction in OD_{600} of the *mtlD*Ω*erm*^{r} mutant (Clearance of cell turbidity) in Triton X-100-induced autolysis assay confirmed that the cell wall of the *mtlD*Ω*erm*^{r} mutant strain is much weaker than those of the WT and complemented strains. **(E)** Visualization of mannitol-induced cell damage of *mtlD*Ω*erm*^{r} knockout *S. aureus* USA300 strain using cell wall-specific wheat germ agglutinin (WTA) fluorescent dye. The indicated bacteria were spotted on BHI agar containing without or with 27.5 mM mannitol and incubated at 37 °C for 48 h. The *S. aureus* cells were harvested and subsequently washed with PBS for 3 times. Bacteria were then resuspended in PBS containing Alexa flour 488 conjugated WGA and incubated for 5 min at room temperature to allow staining of cell-wall of all *S. aureus* cells (3). The free WGA fluorescent dye was removed by 3 times PBS washing. The WGA stained, and washed *S. aureus* cells were resuspended in PBS containing propidium iodide (PI) to visualize membrane-damaged *S. aureus* by assessing the entry and staining with PI. These results confirmed that *mtlD*Ω*erm*^{r} knockout strain lacking *Sa*M1PDH activity were cytolyzed under mannitol treated condition.

References

1. Mani N, Tobin P, Jayaswal RK. 1993. Isolation and characterization of autolysis-defective mutants of *Staphylococcus aureus* created by Tn917-*lacZ* mutagenesis. J Bacteriol 175:1493-9.
2. Bose JL, Lehman MK, Fey PD, Bayles KW. 2012. Contribution of the *Staphylococcus aureus* Atl AM and GL murein hydrolase activities in cell division, autolysis, and biofilm formation. PLoS One 7:e42244.
3. Monteiro JM, Fernandes PB, Vaz F, Pereira AR, Tavares AC, Ferreira MT, Pereira PM, Veiga H, Kuru E, VanNieuwenhze MS, Brun YV, Filipe SR, Pinho MG. 2015. Cell shape dynamics during the staphylococcal cell cycle. Nat Commun 6:8055.
