# Supplementary material for: Targeting Mannitol Metabolism as an Alternative Antimicrobial Strategy Based on the Structure-Function Study of Mannitol-1-Phosphate Dehydrogenase in Staphylococcus aureus
Source: mBio. 2019 Jul 9;10(4):e02660-18. doi: 10.1128/mBio.02660-18 (PMC6623548; doi:10.1128/mBio.02660-18)
Supplement: FIG S4 [file mBio.02660-18-sf004.pdf]

Fig. S4

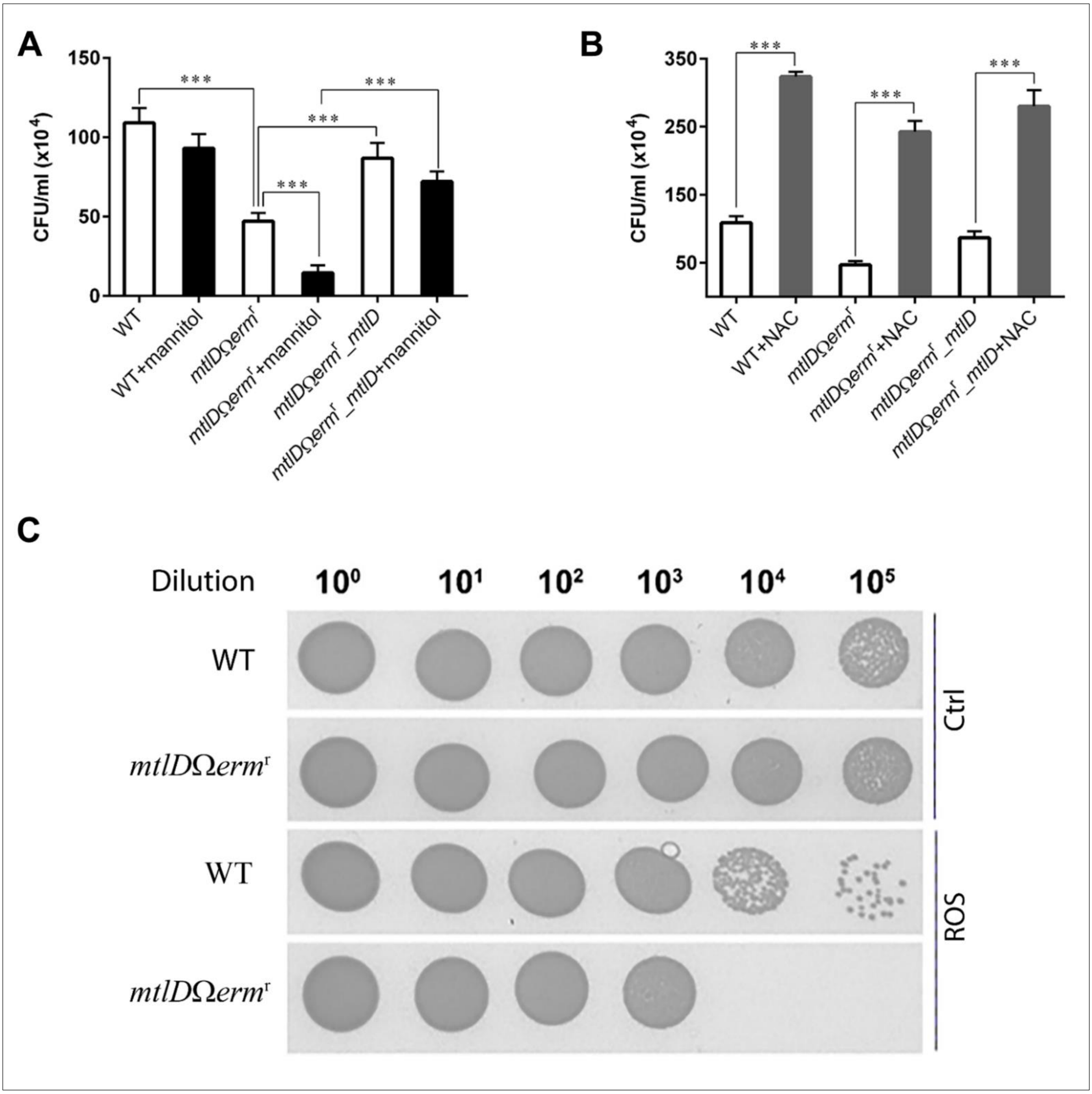

Fig. S4. Role of *Sa*M1PDH with or without mannitol and reactive oxygen species (ROS) in intracellular survival of *S. aureus* strains assessed by modified enzyme protection assay (EPA); and direct assessment of differential sensitivities of *S. aureus* USA300 strains by plate spotting assay. (A-B) Role of *Sa*M1PDH with or without mannitol in intracellular survival of *S. aureus* USA300. Briefly, the overnight cultures of indicated *S. aureus* strains were harvested by centrifugation (4,000 rpm, 5 min) follow by 3-times PBS washing. For infection, *S. aureus* strains were resuspended in DMEM and added to the overnight grown culture of RAW264.7 cells at *moi* of 100 (1-3). The mixture was then incubated at 37 °C in CO_2 incubator for 30 min. Free bacteria were removed by 2-times of PBS washing. The remaining extracellular bacterial cells were killed by 1 unit/ml lysostaphin for 15 min. After removing lysostaphin, the cells were washed with 1,10 phenanthroline and two cycles of PBS washing (Wash-I) (4). The RAW264.7 cells infected with various *S. aureus* strains (host-pathogen complex) in DMEM media in presence or absence of either 2.75 mM mannitol

(+/- mannitol) (**A**) or 5 mM N-Acetyl-L-cysteine (NAC) **(B)** (+/- NAC as a ROS inhibitor) (5). In case of **(B)**, the host-pathogen complexes were further incubated at 37 °C in CO_2 incubator for 6 h to assess the intracellular survival of *S. aureus* strains. In the middle of this incubation, *i.e.* at 3 h, the media DMEM (+/- mannitol) or DMEM (+/- NAC) were removed, and the cells were washed (Wash-II) similarly as wash-I to avoid the extracellular enumeration of escaped *S. aureus* from the infected host cells. At the end of 6 h incubation, the host-pathogen samples were additionally washed (Wash-III) to remove any extracellular *S. aureus* as described for wash 1. The host cells internalized with *S. aureus* strains were lysed, and the CFU was enumerated by dilution plating. The increased susceptibility of the *mtlD*Ω*erm*^{r} knockout strain was observed by ~85 % and ~80 % compared to WT and its complemented strain when cells were treated with 2.75 mM mannitol (**A**). On the contrary, the survival of the Δ*mtlD* knockout strain was increased up to 5-times upon ROS inhibition using NAC, suggested that *Sa*M1PDH contributes to ROS resistance of *S. aureus* during infection (**B**). **(C)** Plate spotting assay showing the differential sensitivities of *S. aureus* USA300 strains. Briefly, overnight cultures of WT and *Sa*M1PDH knockout (*mtlD*Ω*erm*^{r}) *S. aureus* strains were normalized to an OD_{600} of 1.0, 10-fold serially diluted, and spotted (10 µL) onto either BHI agar (Ctrl) or BHI agar containing ROS-generating chemicals (100 µM H_2O_2, 10 µM $FeSO_4$, and 10 µM NaI) (6). After incubation at 37 °C for 24 h, plates were photographed. Images are representative of at least two independent experiments. For clear visualization, images were converted to grayscale mode. The *Sa*M1PDH deficient strain (*mtlD*Ω*erm*^{r} mutant) was found to be 100-times more susceptible against ROS stress than that of WT *S. aureus.*

References

1. Wang X, Wang X, Teng D, Mao R, Hao Y, Yang N, Li Z, Wang J. 2018. Increased intracellular activity of MP1102 and NZ2114 against S*taphylococcus aureus in vitro* and *in vivo*. Sci Rep 8:4204.
2. Hamza T, Li BY. 2014. Differential responses of osteoblasts and macrophages upon *Staphylococcus aureus* infection. BMC Microbiology 14.
3. O'Keeffe KM, Wilk MM, Leech JM, Murphy AG, Laabei M, Monk IR, Massey RC, Lindsay JA, Foster TJ, Geoghegan JA, McLoughlin RM. 2015. Manipulation of autophagy in phagocytes facilitates *Staphylococcus aureus* bloodstream infection. Infect and Immun 83:3445-3457.
4. Kim JH, Chaurasia AK, Batool N, Ko KS, Kim KK. 2019. Alternative enzyme protection assay to overcome the drawbacks of the gentamicin protection assay for measuring entry and intracellular survival of Staphylococci. Infect Immun 87 doi: 10.1128/IAI.00119-19
5. Sun SY. 2010. N-acetylcysteine, reactive oxygen species and beyond. Cancer Biol Ther 9:109-10.
6. Klebanoff SJ. 1982. The iron-H_2O_2-iodide cytotoxic system. J Exp Med 156:1262-7.
