# Supplementary material for: Targeting Mannitol Metabolism as an Alternative Antimicrobial Strategy Based on the Structure-Function Study of Mannitol-1-Phosphate Dehydrogenase in Staphylococcus aureus
Source: mBio. 2019 Jul 9;10(4):e02660-18. doi: 10.1128/mBio.02660-18 (PMC6623548; doi:10.1128/mBio.02660-18)
Supplement: FIG S5 [file mBio.02660-18-sf005.pdf]

Fig. S5

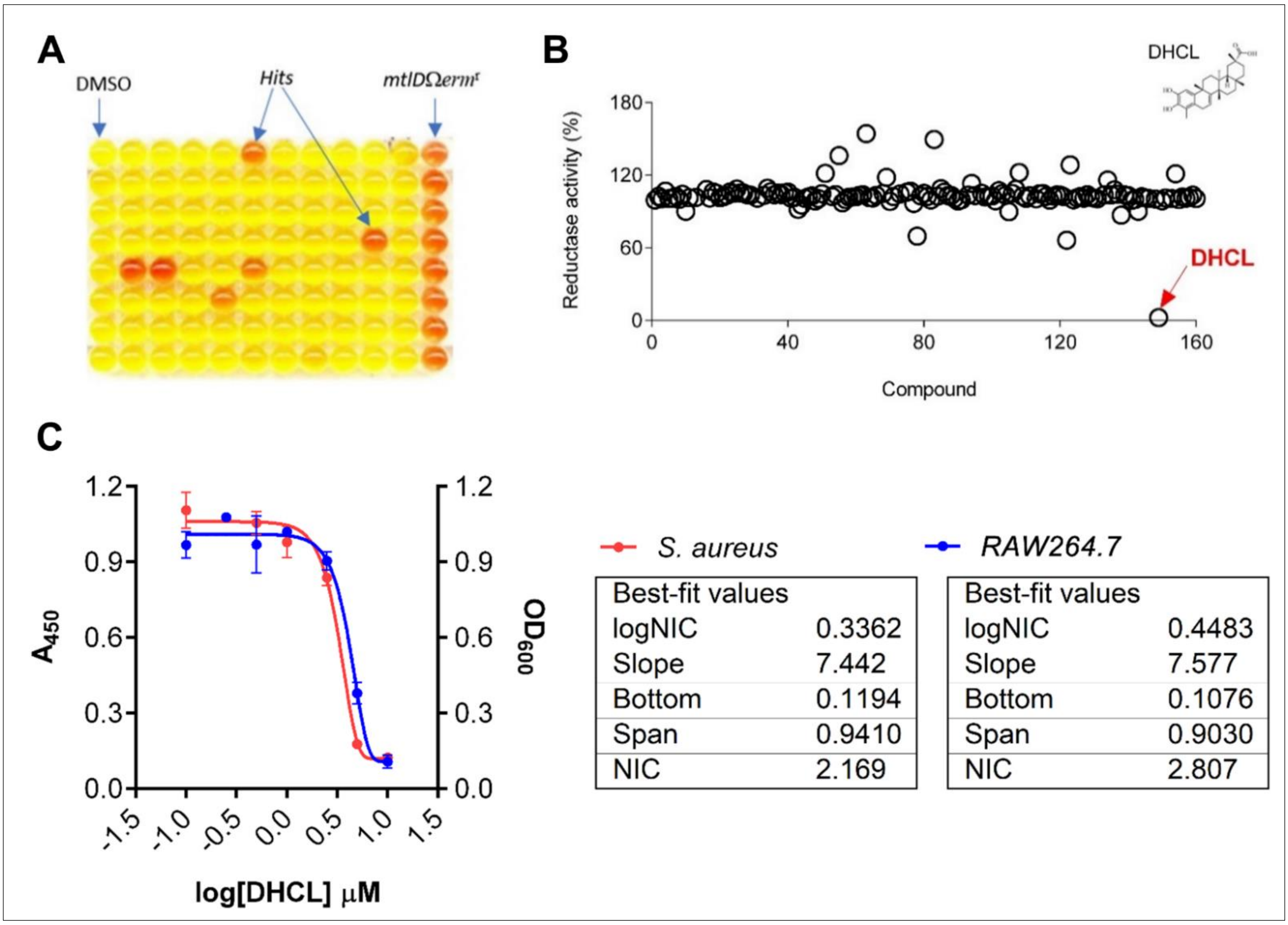

Fig. S5. Functional assays for screening and identifying dihydrocelastrol (DHCL) as a potent inhibitor of *Sa*M1PDH; and its effects on proliferation of *S. aureus* USA300 and RAW264.7 cells. (**A**) Primary screening assay to identify hits. Inhibiting mannitol-metabolizing capacity of WT *S. aureus* USA300 strain was used to screen for *Sa*M1PDH inhibitors. In a mannitol salt broth supplemented with phenol red as a pH indicator, mannitol fermentation of WT *S. aureus* cells generates acidic byproducts that turn broth from red to yellow. Chemicals inhibiting that color change were recognized as hits. WT *S. aureus* cells grown in broth treated with DMSO and *Sa*M1PDH knockout (*mtlD*Ω*erm*^{r}) *S. aureus* cells grown in broth alone were included in each screening plate as a negative and positive control, respectively. Image is representative of screening plates photographed after 6 h incubation at 37 °C with arrows indicating negative controls, positive controls, and hits. (**B**) Secondary screening assay to validate primary hits. Inhibiting *Sa*M1PDH F6P reductase activity *in vitro* was used to validate hits found in **(A)** and identified DHCL as a potent inhibitor of *Sa*M1PDH. Briefly, purified *Sa*M1PDH was incubated with cofactor (NADH), substrate (F6P), and hits found in **(A)** for 5 min at room temperature. Relative reductase activity of *Sa*M1PDH was determined by measuring the absorbance of NADH at 340 nm and calculated as percentages of *Sa*M1PDH activity in treated reaction-mix with hits compared to that in control reaction-mix treated with DMSO. Graph was constructed by plotting reductase activities of *Sa*M1PDH (open circles) against examined hits. Arrow indicates F6P reduction activity of *Sa*M1PDH treated with DHCL, whose chemical structure is shown above graph. **(C)** Effect of DHCL on proliferation of *S. aureus* USA300 and RAW264.7 cells. WT *S. aureus* USA300 and RAW264.7 cells were cultured in appropriate media supplemented with various concentrations of DHCL (0–10 µM). For *S. aureus* cells, DHCL susceptibility was assessed by measuring optical density of bacterial culture at 600 nm (OD_{600}).

For RAW264.7 cells, after addition of a water-soluble tetrazolium salts solution (EZ-Cytox Cell Viability Assay kit, DoGen, Korea) into cultured cells, cell viability was assessed by measuring absorbance at 450 nm (A_{450}). Graph was constructed by plotting either OD_{600} (*S. aureus*) or A_{450} (RAW264.7 cells) values against logarithms of examined DHCL concentrations. Circles (*S. aureus*, red; RAW264.7, blue) represent mean values obtained from at least three independent measurements, whereas whiskers represent one standard deviations of the means. Solid lines (*S. aureus*, red; RAW264.7, blue) represent the best-fit curves determined by nonlinear regression analyses using GraphPad Prism 7 (http://www.graphpad.com) to determine non-inhibitory concentrations (NICs) (1) of DHCL. Nonlinear regression analysis results are summarized in tables.

Reference

1. Lambert RJ, Pearson J. 2000. Susceptibility testing: accurate and reproducible minimum inhibitory concentration (MIC) and non-inhibitory concentration (NIC) values. J Appl Microbiol 88:784-90.
