# Supplementary material for: Targeting Mannitol Metabolism as an Alternative Antimicrobial Strategy Based on the Structure-Function Study of Mannitol-1-Phosphate Dehydrogenase in Staphylococcus aureus
Source: mBio. 2019 Jul 9;10(4):e02660-18. doi: 10.1128/mBio.02660-18 (PMC6623548; doi:10.1128/mBio.02660-18)
Supplement: FIG S6 [file mBio.02660-18-sf006.pdf]

Fig. S6

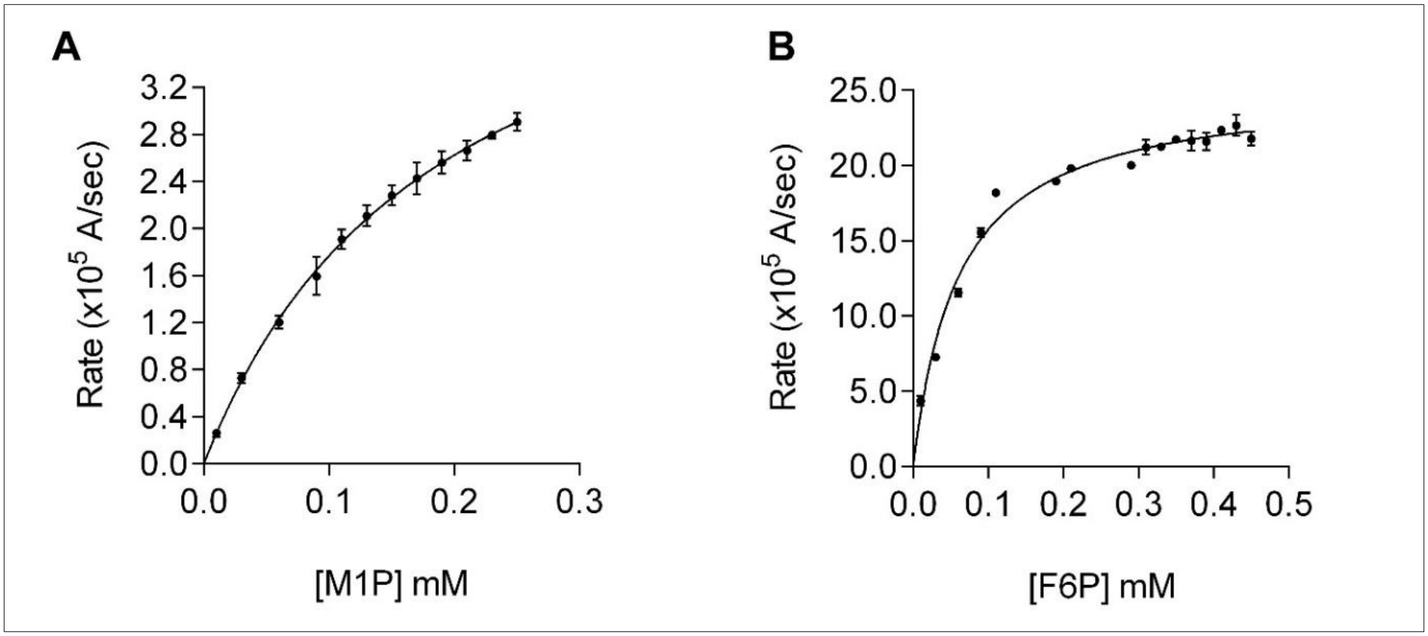

Fig. S6. Determination of Michaelis-Menten kinetic parameters for *Sa*M1PDH oxidoreductase activity. (A) Determination of K_m (substrate concentration that yields a half-maximal velocity) and V_{max} (maximal velocity) values for *Sa*M1PDH mannitol-1-phosphate (M1P) oxidase activity. **(B)** Determination of K_m and V_{max} values for *Sa*M1PDH fructose-6-phosphate (F6P) reductase activity. In a typical reaction, purified *Sa*M1PDH was incubated with cofactor (M1P oxidation, NAD^+; F6P reduction, NADH) and various concentrations of substrate (oxidation reaction, 0–250 µM M1P; reduction reaction, 0–450 µM F6P). At each substrate concentration, initial enzymatic velocity was determined after 1min reaction at 30 °C by measuring change in absorbance of NADH at 340 nm in reaction with substrate compared to that in control reaction without substrate and expressed as absorbance change per second (A/sec). Graphs were constructed by plotting initial enzymatic velocities against substrate concentrations. Dots represent mean values obtained from three independent measurements, whereas whiskers represent one standard deviations of the means. Solid lines represent the best-fit curves determined by nonlinear regression analyses using GraphPad Prism 7 for Mac (http://www.graphpad.com) to calculate K_m and V_{max} values.
