# Supplementary material for: Targeting Mannitol Metabolism as an Alternative Antimicrobial Strategy Based on the Structure-Function Study of Mannitol-1-Phosphate Dehydrogenase in Staphylococcus aureus
Source: mBio. 2019 Jul 9;10(4):e02660-18. doi: 10.1128/mBio.02660-18 (PMC6623548; doi:10.1128/mBio.02660-18)
Supplement: FIG S7 [file mBio.02660-18-sf007.pdf]

Fig. S7

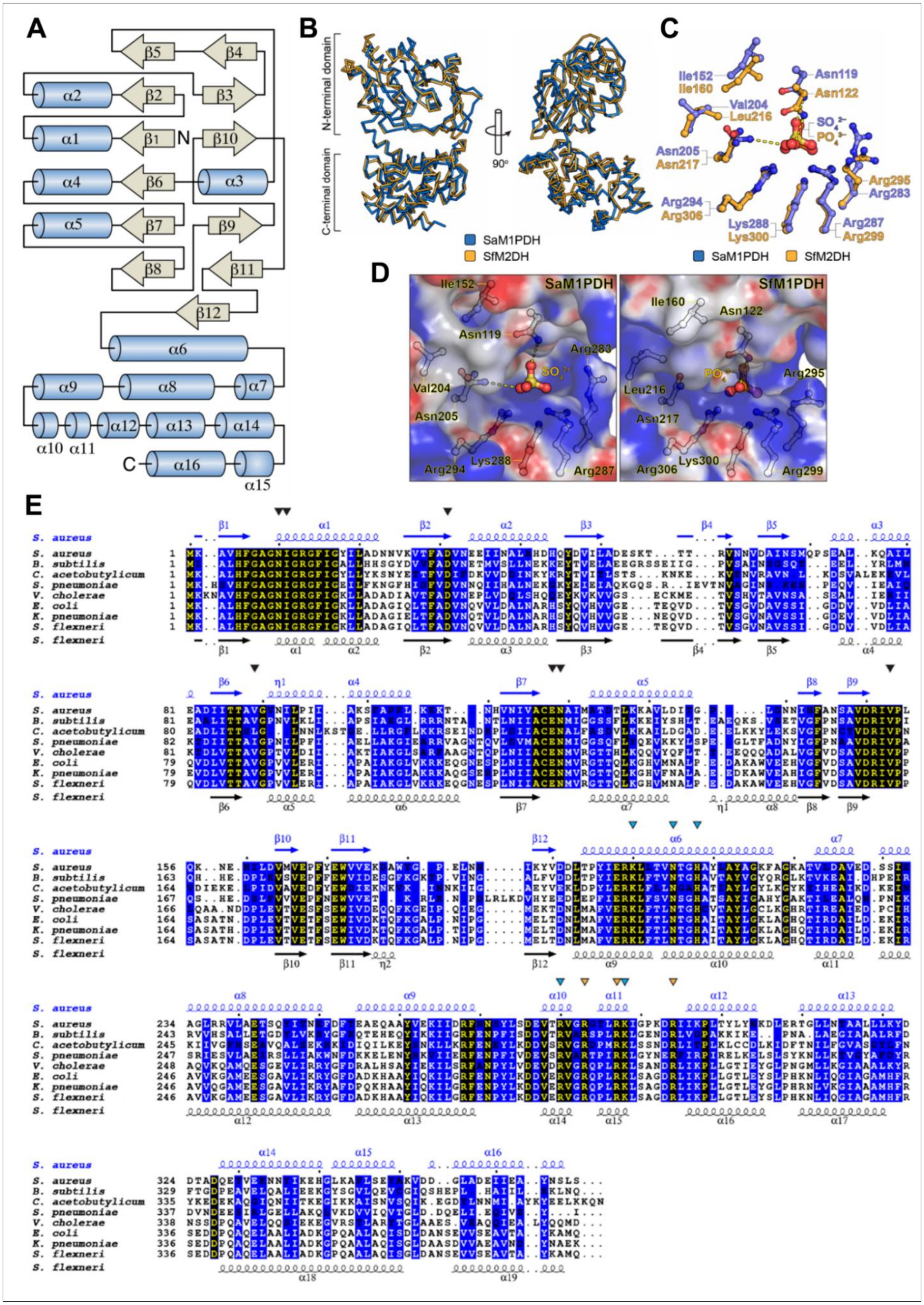

A
B
C
D
E
N-terminal domain
C-terminal domain
90°
SaM1PDH
SfM2DH
Ile152
Ile160
Asn119
Asn122
Val204
Leu216
Asn205
Asn217
Arg294
Arg306
Lys288
Lys300
Arg287
Arg299
Arg295
Arg283
SfM1PDH
Arg287
S. aureus
B. subtilis
C. acetobutylicum
S. pneumoniae
V. cholerae
E. coli
K. pneumoniae
S. flexneri

Fig. S7. Topology diagram of *Sa*M1PDH, its structural comparison with *Shigella flexneri* (*Sf*M1PDH); and multiple sequence alignment of *Sa*M1PDH with its homologs in other bacterial species. (A) Topology diagram showing the overall fold of *Sa*M1PDH wherein cylinders (blue) and arrows (gray) represent α-helices and β-strands, respectively; whereas solid lines (black) represents loops and turns. Figure was adapted from that generated by PDBsum (http://www.ebi.ac.uk/pdbsum). The α-helices and β-strands are numbered in accordance with their orders found in *Sa*M1PDH crystal structure. The N- and C-termini of *Sa*M1PDH are annotated by N and C, respectively. **(B)** Structural superimposition of *Sa*M1PDH (blue) and *Sf*M1PDH (orange; PDB: 3H2Z) in (left) front view and (right) side view. Protein structures are displayed as ribbon diagrams. The N- and C- terminal domains are indicated. **(C)** Structural superimposition of the active sites of *Sa*M1PDH (blue) and *Sf*M1PDH (orange). **(D)** Electrostatic potential surfaces (contouring level of ± 8 kTe^{-1}) of substrate-binding sites of (left) *Sa*M1PDH and (right) *Sf*M1PDH. Structures of *Sa*M1PDH and *Sf*M2DH are superimposed as shown in **(B)**. Electrostatic properties (positive charge, blue; neutral, white; negative, red) were calculated using APBS and PDB2PQR Server (http://nbcr-222.ucsd.edu/pdb2pqr_2.0.0/). In **(C)** and **(D)**, sulfate, phosphate, and key residues in substrate-binding sites are drawn in ball-and-stick models. Dashed lines represent bonds between interacting atoms. Figures were prepared using PyMOL (http://www.pymol.org). **(E)** Multiple sequence alignment of *Sa*M1PDH with its homologs in other bacterial species showing amino acid sequences of M1PDHs collected from four Gram-positive bacteria (*S. aureus*, GenBank: ABD20823.1; *Bacillus subtilis*, GenBank: AQR80339.1; *Clostridium acetobutylicum*, GenBank: AAC12851.1; *Streptococcus pneumoniae*, GenBank: AAK99163.1) and four Gram-negative bacteria (*Vibrio cholerae*, GenBank: AAF96940.1; *Escherichia coli*, GenBank: AAG58744.1; *Klebsiella pneumoniae*, GenBank: AIE00851.1; *Shigella flexneri*, GenBank: AAP19108.1). Strictly conserved residues are colored yellow and boxed in black, whereas highly similar residues are colored white and boxed in blue. Secondary structures of *S. aureus* M1PDH (blue; PDB: 5JNM) and *S. flexneri* M1PDH (black; PDB: 3H2Z) are shown above and below the alignment, respectively. Residues involved in binding to cofactors (NAD^+/NADH), substrate (mannitol), and phosphate moieties of substrates are denoted by black, blue, and orange arrows, respectively. Amino acid sequences of M1PDHs were aligned using T-Coffee Multiple Sequence Alignment Server (http://tcoffee.crg.cat). Alignment figure was adapted from that generated by ESPript 3 (http://espript.ibcp.fr).
