# Supplementary material for: Targeting Mannitol Metabolism as an Alternative Antimicrobial Strategy Based on the Structure-Function Study of Mannitol-1-Phosphate Dehydrogenase in Staphylococcus aureus
Source: mBio. 2019 Jul 9;10(4):e02660-18. doi: 10.1128/mBio.02660-18 (PMC6623548; doi:10.1128/mBio.02660-18)
Supplement: TABLE S1 [file mBio.02660-18-st001.pdf]

Table S1. Presence (○) and absence (×) of M1PDH and M2DH in different *S. aureus* strains, plant, and fungi.

Strains/organisms	M1PDH	M2DH	GenBank Accession No. or References
***S. aureus* MRSA252**	○	×	BX571856.1
***S. aureus* 13420**	○	×	CP021141.1
***S. aureus* 0201753-2**	○	×	CP021352.1
***S. aureus* AR_0470**	○	×	CP029653.1
***S. aureus* CFSAN007896**	○	×	CP020467.1
***S. aureus* CFSAN018749**	○	×	CP028190.1
***S. aureus* NRS143**	○	×	CP026071.1
***S. aureus* Seattle 1945 isolate G478**	○	×	CP021907.1
***S. aureus* SJTUF_J27**	○	×	CP019117.1
***S. aureus* FORC_001**	○	×	CP009554.1
***S. aureus* ATCC 25923**	○	×	CP009361.1
***S. aureus* NRS484**	○	×	CP026066.1
***S. aureus* CFSAN007847**	○	×	CP017684.1
***S. aureus* TCH60**	○	×	CP002110.1
***S. aureus* Tager 104**	○	×	CP012409.1
***S. aureus* 6850**	○	×	CP006706.1
***S. aureus* LGA251**	○	×	FR821779.1
***S. aureus* NRS153**	○	×	CP026067.1
***S. aureus* 93b_S9**	○	×	CP010952.1
***S. aureus* SA40TW**	○	×	CP013182.1
Platymonas subcordiformis	○	○	Reference (1)
Aspergillus fumigatus	○	○	Reference (2)

References

1. Richter DFE, Kirst GO. 1987. d-Mannitol dehydrogenase and d-mannitol-1-phosphate dehydrogenase in *Platymonas subcordiformis*: some characteristics and their role in osmotic adaptation. Planta 170:528-534.
2. Krahulec S, Armao GC, Klimacek M, Nidetzky B. 2011. Enzymes of mannitol metabolism in the human pathogenic fungus *Aspergillus fumigatus* - kinetic properties of mannitol-1-phosphate 5-dehydrogenase and mannitol 2-dehydrogenase, and their physiological implications. Febs Journal 278:1264-1276.
