# Supplementary material for: Targeting Mannitol Metabolism as an Alternative Antimicrobial Strategy Based on the Structure-Function Study of Mannitol-1-Phosphate Dehydrogenase in Staphylococcus aureus
Source: mBio. 2019 Jul 9;10(4):e02660-18. doi: 10.1128/mBio.02660-18 (PMC6623548; doi:10.1128/mBio.02660-18)
Supplement: TEXT S1 [file mBio.02660-18-s0001.pdf]

Supplemental Methods

Targeting mannitol metabolism as an alternative antimicrobial strategy based on the structure-function studies of mannitol-1-phosphate dehydrogenase in *Staphylococcus aureus*

Running Head:

M1PDH as a novel antimicrobial target

Thanh Nguyen[a], Truc Kim[a], Hai Minh Ta[a], Yeo Won Sik[b], Jongkeun Choi[c], Pushpak Mizar[d], Seung Seo Lee[d], Taeok Bae[b], Akhilesh Kumar Chaurasia[a]*, and Kyeong Kyu Kim[a,e]*

[a]Department of Molecular Cell Biology, Institute for Antimicrobial Research and Therapeutics, Sungkyunkwan University School of Medicine, Suwon 16419, Korea

[b]Department of Microbiology and Immunology, Indiana University School of Medicine-Northwest, Gary, IN 46408, USA

[c]Department of Cosmetic Science, Chungwoon University, Daehakgil-25, Hongseong-eup, Hongseong-gun, Chungnam, 350-701, Korea

[d]School of Chemistry, University of Southampton, Southampton SO17 1BJ, UK

[e]Samsung Biomedical Research Institute, Samsung Advanced Institute for Health Sciences and Technology, Samsung Medical Center, Sungkyunkwan University School of Medicine, Seoul 06351, Korea

*To whom all correspondence should be addressed:

Tel: 82-31-299-6136

Fax: 82-31-299-6159

E-mail: chaurasia@skku.edu, kyeongkyu@skku.edu

1. Construction of *Sa*M1PDH-expressing plasmid in *E. coli*

The *mtlD* gene encoding for *Sa*M1PDH was PCR amplified from *S. aureus* RN4220 genomic DNA (1) using the primer pair *mtlD*_fwd/*mtlD*_rev (Table S4). The PCR product was then cloned at the *Bam*HI/*Xho*I sites of the plasmid pVFT1S (Korean Patent No. 1020050051893), a vector overexpressing protein under the control of an IPTG-inducible T7 promoter. The resultant plasmid, pVFT1S_*mtlD,* incorporates a His_6 tag followed by a TEV protease recognition site into the N-terminus of *Sa*M1PDH.

For introducing the point mutations *R283S*, *R287S*, and *R294F* into the plasmid pVFT1S_*mtlD*, the QuikChange Site-Directed Mutagenesis Kit (Stratagene, USA) was used as per the manufacturer's instructions, generating the plasmids pVFT1S_*R283S*, pVFT1S_*R287S*, and pVFT1S_*R294F*. The mutagenesis primers are listed in Table S4.

2. Protein expressions and purifications

Escherichia coli BL21(DE3) cells harboring the plasmids expressing WT or mutant *Sa*M1PDH were cultured in the lysogenic broths at 37 °C until the optical density at 600 nm (OD_{600}) reached 0.4−0.6. The culture was cooled to 18 °C, and the expression of *Sa*M1PDH was induced by addition of 0.5 mM IPTG. After 24 h, cells were harvested by centrifugation at 6,000 × g for 10 min at 4 °C, resuspended in buffer A (50 mM Tris-HCl pH 8.0, 0.5 M NaCl, and 20 mM imidazole), and disrupted by sonication on ice. After a centrifugation at 48,000 × g for 1 h at 4 °C, the cell-free extracts were loaded onto a 5-mL Ni-NTA column (GE Healthcare, USA), and bound proteins were eluted by a gradient of 0.02–1.0 M imidazole in buffer A. The elution fractions containing *Sa*M1PDH were buffer exchanged to 25 mM HEPES pH 7.5 using a HiLoad 16/60 Superdex 200 prep-grade column (GE Healthcare, USA). Proteins were then concentrated and stored at –80 °C until use.

For the preparation of selenomethionine-substituted *Sa*M1PDH (SeMet-*Sa*M1PDH), *E. coli* BL834(DE3) harboring plasmid pVFT1S_*mtlD* was grown in M9 medium supplemented with 100 mg/L L-selenomethionine (Calbiochem, USA). The SeMet-*Sa*M1PDH protein was expressed and purified according to the same procedure as for the native protein, except 5 mM DTT was added to all buffers to protect the selenium from oxidation.

3. Complementation of *Sa*M1PDH knockout (*mtlD*Ω*erm*^{r}) *S. aureus* strain with wild-type and mutant alleles

The WT *mtlD* gene was amplified from *S. aureus* USA300 genomic DNA using the forward primer *mtlD*_comp._fwd containing the P_{gapA} ribosome-binding site and the reverse primer *mtlD*_comp._rev (Table S4). Using the same primer pair, the point-mutant *mtlD* genes at three Arg residues (*R283S*, *R287S*, and *R294F*) were individually PCR amplified from the template plasmids pVFT1S_*R283S*, pVFT1S_*R287S*, and pVFT1S_*R294F*. The PCR-amplified WT and Arg mutant *mtlD* genes (*R283S*, *R287S*, and *R294F*) were restriction digested with *Kpn*I/*Eco*RI and cloned at the same sites of the tetracycline-inducible vector pRMC2 (2). The preparations of electrocompetent *S. aureus* cells and the electroporation of plasmids into these cells were performed following the procedure described by Grosser and Richardson (3). The recombinant plasmids bearing the WT (pRMC2_*mtlD*) and point-mutant *mtlD* genes (pRMC2_*R283S*, pRMC2_*R287S*, and pRMC2_*R294F*) (Table S5) were introduced into RN4220, followed by electroporation into the *Sa*M1PDH knockout (*mtlD*Ω*erm*^{r}) *S. aureus* USA300 strain to create the complementation strains (Table S6). The pRMC2 empty vector was electroporated into the WT and *Sa*M1PDH knockout (*mtlD*Ω*erm*^{r}) *S. aureus* strains to create the empty vector controls during the physiological studies (Table S6).

4. Determination of the intracellular mannitol levels using a gas chromatography-mass spectrometry (GC-MS) analysis

To examine the accumulations of the intracellular mannitol in the WT and *Sa*M1PDH knockout (*mtlD*Ω*erm*^{r}) *S. aureus* strains using the GC-MS analyses, the samples were prepared as described by Kenny *et al.* (4) with the following modifications. Briefly, after a 6-h culture in the BHI media at 37 °C, the *S. aureus* cells were harvested by centrifugation and washed four times with PBS. Following a flash freezing in liquid nitrogen, the cells were lysed by lysostaphin treatment as described above for the colorimetric assay of mannitol. For a complete lysis, the cells were subjected to the sonications on ice on a Vibra-Cell Ultrasonic Processor VC750 (Sonics, USA). The absolute methanol was then added into the cell lysates to a final concentration of 70%, and the mixtures were incubated at room temperature for 15 min. After a centrifugation to remove the precipitates, the supernatants were lyophilized on a FreeZone 4.5-Litter Benchtop Freeze Dryer (Labconco, USA). The sample derivatizations and the GC-MS analyses followed the procedure described by Kenny *et al.* (4) with a minor modification in which 5 μL of adonitol was added to each sample as an internal standard prior to trimethylsilylation. After comparing spectra of chromatogram peaks with an electron impact mass spectrum library NIST08 (NIST, USA) followed by normalization, relative abundance of mannitol in each sample was determined by absorbance peak of mannitol in chromatography retention profiles. Data points (WT, diamond; knockout, square) represent absorbance of mannitol recorded at each chromatographic retention profiles.

5. *S. aureus* colony morphology assays

After an overnight culture in the BHI media at 37 °C, *S. aureus* cells were harvested by centrifugation at 783 × *g* for 15 min at 4 °C and washed thrice with PBS. To examine the effect of mannitol on the colony morphologies of different *S. aureus* strains, bacterial suspensions were

diluted to an OD_{600} of 1.0, 10-fold serially diluted, and either spotted (10 µL) or plated (100 µL) onto the BHI agar containing 27.5 mM mannitol. After an incubation at 37 °C, the plates were photographed at 48 h and 72 h. The relative diameters of 15–25 colonies that were randomly selected from each *S. aureus* strains were measured from the 48-h photographs using the ImageJ software (5).

6. Membrane permeabilization assay

Bacteria were spotted on BHI agar containing without or with 27.5 mM mannitol and incubated at 37 °C for 48 h. The cells were harvested and subsequently washed with PBS for 3 times. Bacterial cells were then resuspended in PBS containing 2 µg/ml Alexa flour 488 conjugated Wheat Germ Agglutinin (WGA, Invitrogen, USA) and incubated for 5 min at room temperature to allow staining of cell-wall of all *S. aureus* cells. The free WGA fluorescent dye was removed by 3 times PBS washing. The WGA stained *S. aureus* cells were resuspended in PBS containing 10 µg/ml propidium iodide (PI, ThermoFisher Scientific, USA) and incubated for 15 min at room temperature to visualize membrane-damaged *S. aureus* due to entry and red fluorescent staining of bacterial cells with PI. The bacterial cells were subjected to confocal imaging and analysis using Leica Application Suite X (LAS X, Leica Microsystems)

7. Mannitol catabolism assay

Indicated *S. aureus* strains were cultured under static culture conditions in mannitol (55 mM) phenol red broth for 24 h at 37 °C in a 24-well plate before being photographed. The phenol red color turned into yellow which indicates mannitol catabolism.

8. Screening assays to identify the small-molecule inhibitors of *Sa*M1PDH

To identify the inhibitor of *Sa*M1PDH from a Natural Product Collection (MicroSource Discovery Systems, USA), two screening assays were developed: (*i*) a primary screening assay to identify hits and (*ii*) a secondary screening assay to validate the hits. In the primary screening

assay, inhibiting the mannitol-metabolizing capacity of WT *S. aureus* USA300 strain was used to screen for *Sa*M1PDH inhibitors. In a mannitol salt broth supplemented with phenol red as a pH indicator, the mannitol fermentation of WT *S. aureus* cells generates acidic byproducts that turn the broth from red to yellow. Any compounds that inhibit that color change were recognized as hits. Prior to the assay, the WT *S. aureus* USA300 cells were grown in the BHI media at 37 °C overnight, harvested by centrifugation, and washed thrice with PBS. The cells were then diluted to an OD_{600} of 0.05 in the Mannitol Salt Broth (10 g/L peptone, 1 g/L beef extract, 75 g/L NaCl, and 10 g/L D-mannitol) containing phenol red (25 mg/L). To each well of the 96-well plates were added 200 µL of the *S. aureus* cell suspension and 1 µL of each natural compound stock solution to give a final compound concentration of 10 µM. The WT *S. aureus* cells grown in the broth containing 0.5% DMSO and the *Sa*M1PDH knockout (*mtlD*Ω*erm*^{r}) *S. aureus* cells grown in the broth alone were included in each screening plate as a negative and positive control, respectively. Following an incubation at 37 °C for 6 h, the plates were photographed, the color change of the broth was examined, and the hit compounds were selected.

In the secondary screening assay, inhibiting *Sa*M1PDH F6P reduction activity *in vitro* was used to validate the hits selected from the primary screening. In the assay, similar reactions were done as described above for examining the time-dependent F6P reductase activity of *Sa*M1PDH with the three modifications as follows: (*i*) NADH was used in the reaction at a final concentration of 250 µM, (*ii*) each hit compound selected from the primary screening was added into the reaction at a final concentration of 10 µM, and (*iii*) the absorbance of NADH at 340 nm was measured after incubating the reaction at room temperature for 5 min. Following examining the change in the absorbance of NADH at 340 nm for each reaction, any compounds that cause no reduction in the absorbance of NADH were selected as the potent inhibitors of *Sa*M1PDH and subjected to further experimental assay studies to validate their inhibitory activities.

9. Cell viability assays

To examine the inhibitory effect of dihydrocelastrol (DHCL) on the growth of WT *S. aureus* USA300, a cell growth assay was done as described above for those under the abiotic stresses with minor modifications. Briefly, the diluted overnight culture of WT *S. aureus* was progressively grown in the BHI media supplemented with various concentrations of DHCL (0–10 μM) on a 96-well plate. After a 12-h incubation at 37 °C, the bacterial OD_{600} values at each DHCL concentration were measured on the Infinite M200 Microplate Reader (Tecan, USA).

To examine the inhibitory effect of DHCL on the proliferation of the RAW264.7 cells, an EZ-Cytox Cell Viability Assay kit (DoGen, Korea) was used according to the manufacturer's instructions. Briefly, before being treated with DHCL, the RAW264.7 cells were seeded in the low-glucose Dulbecco's Modified Eagle's Medium (DMEM; Welgene, Korea) supplemented with 10% fetal bovine serum (FBS; Welgene, Korea) and allowed to grow at 37 °C for 24 h on a 96-well plate in a CO_2 incubator (5% CO_2). Various concentrations of DHCL (0–10 μM) were then supplemented into the RAW264.7 cell cultures, and the macrophages were allowed to grow for an additional 24 h at 37 °C in the CO_2 incubator. For the viability assays, 10 μL of the EZ-Cytox solution was added to each well of the culture plate, and the mixtures were incubated for 2 h at 37 °C in the CO_2 incubator. Macrophages viabilities were examined by measuring the absorbance at 450 nm (A_{450}) at each DHCL concentration on the Infinite M200 Microplate Reader (Tecan, USA).

The non-inhibitory concentrations (NICs) of DHCL on the proliferation of WT *S. aureus* USA300 and the RAW264.7 cells were determined by a nonlinear regression analysis using the GraphPad Prism 7 for Mac (http://www.graphpad.com). The nonlinear regression analysis results are summarized in Fig. S6.

10. Reactive oxygen species (ROS) susceptibility assay

The ROS susceptibilities of *S. aureus* strains were examined according to the method described by Klebanoff *et al*. (6) with the following modifications. Briefly, after an overnight culture in the BHI media at 37 °C, *S. aureus* cells were harvested by centrifugation at 783 × *g* for 5 min at 4 °C and washed thrice with PBS. Bacterial suspensions were then diluted to an OD_{600} of 1.0, 10-fold serially diluted, and spotted (10 µL) onto BHI agar containing ROS-generating chemicals (100 µM H_2O_2, 10 µM $FeSO_4$, and 10 µM NaI). After an incubation at 37 °C for 24 h, the plates were photographed.

11. Molecular docking of substrates and inhibitor in the active site of *Sa*M1PDH

Prior to the molecular docking, the structures of *Sa*M1PDH (PDB: 5JNM), mannitol-1-phosphate (M1P, PubChem CID: 130418), fructose-6-phosphate (F6P, PubChem CID: 69507), and dihydrocelastrol (DHCL, PubChem CID: 10411574) were prepared using the UCSF Chimera software (7). The conserved residues involved in sulfate and mannitol binding (Lys200, Asn205, His208, Arg280, Arg283, Arg287, Lys288, and Arg294) were used to guide the placements of the ligands and inhibitors into the prepared *Sa*M1PDH structure during the molecular docking studies and structural refinements. The flexible ligand docking, and energy minimization were done using the Dock 6 v.6.8 (8) following the software instructions (http://dock.compbio.ucsf.edu). The top-score models were selected and further validated using the UCSF Chimera (7) and LigPlot$^+$ v.2.1 (9).

12. Quantitative real-time PCR

An overnight *S. aureus* culture was diluted to an OD_{600} of 0.05 in the unmodified BHI media and allowed to grow at 37 °C with agitation until mid-log phase. After being harvested by centrifugation at 783 × *g* for 15 min and washed thrice with PBS, *S. aureus* cells were resuspended in either unmodified BHI media or modified BHI media mimicking abiotic stresses. Following a 30-min incubation at 37 °C with agitation, bacterial suspension was immediately mixed by

vortexing with two volumes of an RNAprotect Bacteria Reagent (Qiagen, USA). Bacteria were then harvested by centrifugation at 783 × *g* for 15 min and stored at –80 °C until use.

Prior to RNA purification, *S. aureus* cell pellet was resuspended in 200 µL of a lysis buffer (10 mM Tris-HCl pH 8.0, 1 mM EDTA, 15 mg/mL lysozyme, and 1 unit of lysostaphin) and incubated at 37 °C for 30 min. Total *S. aureus* RNA was then isolated using an RNAeasy Mini Kit (Qiagen, USA) following manufacturer's protocol. Two micrograms of purified RNA were treated with an amplification grade DNase I and reverse transcribed using an RNA to cDNA EcoDry Premix (Random Hexamers; Takara, Japan) according to manufacturers' instructions. The qRT-PCR was conducted on a CFX Connect Real-Time PCR Detection System (Bio-Rad, USA). A typical 15-µL amplification reaction contained 20 ng cDNA, 300 nM of each of forward and reverse primers, and 1× iTaq Universal SYBR Green Supermix (Bio-Rad, USA). A typical thermal cycling reaction consisted of an initial polymerase activation and DNA denaturation at 95 °C for 30 sec followed by 40 cycles of denaturation at 95 °C for 5 sec and annealing/extension at 60 °C for 30 sec. To validate the amplification specificities of the qRT-PCR primers (Table S4), the qRT-PCR products were subjected to a melting curve analysis using a built-in program of the thermal cycler followed by agarose electrophoresis. The relative abundance of the transcripts was normalized against those of *rpoB* and *rho* genes, and fold change between samples was calculated by the ΔCt method using a CFX Manager software (Bio-Rad, USA).

References

1. Peng HL, Novick RP, Kreiswirth B, Kornblum J, Schlievert P. 1988. Cloning, characterization, and sequencing of an accessory gene regulator (agr) in *Staphylococcus aureus*. J Bacteriol 170:4365-72.
2. Corrigan RM, Foster TJ. 2009. An improved tetracycline-inducible expression vector for *Staphylococcus aureus*. Plasmid 61:126-9.
3. Grosser MR, Richardson AR. 2016. Method for Preparation and Electroporation of *S. aureus* and *S. epidermidis*. Methods Mol Biol 1373:51-7.
4. Kenny JG, Moran J, Kolar SL, Ulanov A, Li Z, Shaw LN, Josefsson E, Horsburgh MJ. 2013. Mannitol Utilisation is Required for Protection of *Staphylococcus aureus* from Human Skin Antimicrobial Fatty Acids. Plos One 8.
5. Rueden CT, Schindelin J, Hiner MC, DeZonia BE, Walter AE, Arena ET, Eliceiri KW. 2017. ImageJ2: ImageJ for the next generation of scientific image data. Bmc Bioinformatics 18.
6. Klebanoff SJ. 1982. The iron-H2O2-iodide cytotoxic system. J Exp Med 156:1262-7.
7. Pettersen EF, Goddard TD, Huang CC, Couch GS, Greenblatt DM, Meng EC, Ferrin TE. 2004. UCSF Chimera--a visualization system for exploratory research and analysis. J Comput Chem 25:1605-12.
8. Lang PT, Brozell SR, Mukherjee S, Pettersen EF, Meng EC, Thomas V, Rizzo RC, Case DA, James TL, Kuntz ID. 2009. DOCK 6: combining techniques to model RNA-small molecule complexes. RNA 15:1219-30.
9. Wallace AC, Laskowski RA, Thornton JM. 1995. LIGPLOT: a program to generate schematic diagrams of protein-ligand interactions. Protein Eng 8:127-34.
