# Supplementary material for: Targeting Mannitol Metabolism as an Alternative Antimicrobial Strategy Based on the Structure-Function Study of Mannitol-1-Phosphate Dehydrogenase in Staphylococcus aureus
Source: mBio. 2019 Jul 9;10(4):e02660-18. doi: 10.1128/mBio.02660-18 (PMC6623548; doi:10.1128/mBio.02660-18)
Supplement: TABLE S2 [file mBio.02660-18-st002.pdf]

Table S2. Primers, plasmids and bacterial strains used in this study.

A. Primers used in this study

**Primer name**	**Sequence (5'→3')**[a]	**Purpose**
*mtlD*_fwd	TAGGATCCATGAAAGCAGTTCACTTTGGTG (*Bam*HI)	cloning of *S. aureus mtlD* gene into plasmid pVFT1S
*mtlD*_rev	GTCTCGAGTTATGAAAGTGAATTGTACGCTTCA (*Xho*I)	
*mtlD*_comp._fwd	ATTATAGGTACC*AGAA****AGGAGG****TAAACAT*ATGAAAGCAGTTCACTTTG (*Kpn*I)	cloning of *mtlD* wild type and mutants into plasmid pRMC2
*mtlD*_comp._rev	AATAATGAATTCTTATGAAAGTGAATTGTAC (*Eco*RI)	
*R283S*_fwd	CACGTGTCGGAAGCGGTACATTACGTAAAATTGGCCC	introducing *R283S* mutation into *mtlD*
*R283S*_rev	GGGCCAATTTTACGTAATGTACCGCTTCCGACACGTG	
*R287S*_fwd	GTCGGACGAGGTACATTAAGTAAAATTGGCCCTAAAG	introducing *R287S* mutation into *mtlD*
*R287S*_rev	CTTTAGGGCCAATTTTACTTAATGTACCTCGTCCGAC	
*R294F*_fwd	GCCCTAAAGATTTCATTATAAAAC	introducing *R294F* mutation into *mtlD*
*R294F*_rev	GGTTTTATAATGAAATCTTTAGGGC	
*mtlA*_fwd	AGCAATTCCTCATGGCACAG	qRT-PCR for *mtlA* gene
*mtlA*_rev	TTACCAGCAATTCCCACGAC	
*mtlR*_fwd	TCGTTTGCAGCAGTGGTATG	qRT-PCR for *mtlR* gene
*mtlR*_rev	ATCGCTGACTGATGCTTGTG	
*mtlF*_fwd	AAAGGCACTGCAAAAGCAAC	qRT-PCR for *mtlF* gene
*mtlF*_rev	TGCAACACCAGTCATTCCAC	
*mtlD*_fwd	TGGCTTACGCCGTGTTTTAG	qRT-PCR for *mtlD* gene
*mtlD*_rev	AATGTACCTCGTCCGACACG	
*rho*_fwd	AACGTTTGACGAACCACCAG	qRT-PCR for
*rho*_rev	ATAAGCGCGTGCTAATCTCG	*rho* gene
*rpoB*_fwd	TGAAGAGAACGGCGTTGAG	qRT-PCR for *rpoB* gene
*rpoB*_rev	CCAACTGCAACGATTGGAC	

[a] The restriction sites are underlined, and the corresponding restriction endonucleases are shown in parentheses. The Shine-Dalgarno sequence of *gapA* promoter is in bold.

B. Plasmids used in this study.

Plasmids	Descriptions	References
pVFT1S	*km*^{r}; expression vector tagging His_6 to the N-terminus of proteins	Korean Patent 1020050051893
pVFT1S_*mtlD*	*km*^{r}; pVFT1S expressing *Sa*M1PDH wild type	This study
pVFT1S_*R283S*	*km*^{r}; pVFT1S expressing *Sa*M1PDH *R283S* mutant	This study
pVFT1S_*R287S*	*km*^{r}; pVFT1S expressing *Sa*M1PDH *R287S* mutant	This study
pVFT1S_*R294F*	*km*^{r}; pVFT1S expressing *Sa*M1PDH *R294* mutant	This study
pRMC2	*amp*^{r}, *cm*^{r}; expression vector under control of tetracycline inducible $P_{xyl/tetO}$	Reference (1)
pRMC2_*mtlD*	*amp*^{r}, *cm*^{r}; pRMC2 expressing *Sa*M1PDH wild type	This study
pRMC2_*R283S*	*amp*^{r}, *cm*^{r}; pRMC2 expressing *Sa*M1PDH *R283S* mutant	This study
pRMC2_*R287S*	*amp*^{r}, *cm*^{r}; pRMC2 expressing *Sa*M1PDH *R287S* mutant	This study
pRMC2_*R294F*	*amp*^{r}, *cm*^{r}; pRMC2 expressing *Sa*M1PDH *R294F* mutant	This study

C. Bacterial strains used in this study.

Strains	Descriptions	Sources or References
Escherichia coli		
DH5α	Strain for cloning and plasmid amplification	Invitrogen, USA
BL21(DE3)	Strain for protein overexpression	Novagen, USA
B834(DE3)	Strain for selenomethionine-substituted protein overexpression	Novagen, USA
Staphylococcus aureus		
RN4220	Restriction-deficient strain of NCTC8325	Reference (2)
RN_RMC2	*cm*^{r}, *amp*^{r}; RN4220 bearing plasmid pRMC2	This study
RN_*mtlD*	*amp*^{r}, *cm*^{r}; RN4220 bearing plasmid pRMC2_*mtlD*	This study
RNR283S	*amp*^{r}, *cm*^{r}; RN4220 bearing plasmid pRMC2_*R283S*	This study
RNR287S	*amp*^{r}, *cm*^{r}; RN4220 bearing plasmid pRMC2_*R287S*	This study
RNR294F	*amp*^{r}, *cm*^{r}; RN4220 bearing plasmid pRMC2_*R294F*	This study
WT SAUSA300	JE2, wild-type epidemic community-associated methicillin-resistant *S. aureus* isolate USA300 LAC	Nebraska library (3)
*mtlA*Ω*erm*^{r}	NE929, *erm*^{r}; JE2 bearing *mtlA*::*Tn917*	Nebraska library (3)
*mtlR*Ω*erm*^{r}	NE837, *erm*^{r}; JE2 bearing *mtlR*::*Tn917*	Nebraska library (3)
*mtlF*Ω*erm*^{r}	NE1737, *erm*^{r}; JE2 bearing *mtlF*::*Tn917*	Nebraska library (3)
*mtlD*Ω*erm*^{r}	NE1263, *erm*^{r}; JE2 bearing *mtlD*::*Tn917*	Nebraska library (3)
WT SAUSA300_pRMC2	*cm*^{r}, *amp*^{r}; WT SAUSA300 bearing plasmid pRMC2	This study
*mtlD*Ω*erm*^{r}_pRMC2	*erm*^{r}, *amp*^{r}, *cm*^{r}; NE1263 bearing plasmid pRMC2	This study
*mtlD*Ω*erm*^{r}_*mtlD*	*erm*^{r}, *amp*^{r}, *cm*^{r}; NE1263 bearing plasmid pRMC2_*mtlD*	This study

*mtlD*Ω*erm*^{r}*_R283S*	*erm*^{r}, *amp*^{r}, *cm*^{r}; NE1263 bearing plasmid pRMC2_*R283S*	This study
*mtlD*Ω*erm*^{r}*_R287S*	*erm*^{r}, *amp*^{r}, *cm*^{r}; NE1263 bearing plasmid pRMC2_*R287S*	This study
*mtlD*Ω*erm*^{r}*_R294F*	*erm*^{r}, *amp*^{r}, *cm*^{r}; NE1263 bearing plasmid pRMC2_*R294F*	This study
*mtlA*Ω*erm*^{r}*_mtlD*	*erm*^{r}, *amp*^{r}, *cm*^{r}; NE929 bearing plasmid pRMC2_*mtlD*	This study
*mtlR*Ω*erm*^{r}*_mtlD*	*erm*^{r}, *amp*^{r}, *cm*^{r}; NE837 bearing plasmid pRMC2_*mtlD*	This study
*mtlF*Ω*erm*^{r}*_mtlD*	*erm*^{r}, *amp*^{r}, *cm*^{r}; NE1737 bearing plasmid pRMC2_*mtlD*	This study
WT_pRMC2	*cm*^{r}; WT SAUSA300 bearing plasmid pRMC2	This study

References

1. Corrigan RM, Foster TJ. 2009. An improved tetracycline-inducible expression vector for *Staphylococcus aureus*. Plasmid 61:126-9.
2. Peng HL, Novick RP, Kreiswirth B, Kornblum J, Schlievert P. 1988. Cloning, characterization, and sequencing of an accessory gene regulator (agr) in *Staphylococcus aureus*. J Bacteriol 170:4365-72.
3. Fey PD, Endres JL, Yajjala VK, Widhelm TJ, Boissy RJ, Bose JL, Bayles KW. 2013. A genetic resource for rapid and comprehensive phenotype screening of nonessential *Staphylococcus aureus* genes. MBio 4:e00537-12.
